# Drying Very Preterm Infants Before Plastic Wrapping at Birth: A Randomized Clinical Trial

**DOI:** 10.1001/jamanetworkopen.2025.56902

**Published:** 2026-03-03

**Authors:** Francesco Cavallin, Nicoletta Doglioni, Francesco Maria Risso, Carlo Bernardo Monari, Salvatore Aversa, Stefania Troiani, Nadia Battajon, Sabino Moschella, Paolo Ernesto Villani, Stefania Vedovato, Danuska Maiorca, Simonetta Frezza, Gianluca Lista, Nicola Laforgia, Isabella Mondello, Irene Sibona, Alex Staffler, Simone Pratesi, Giulia Paviotti, Giuseppe De Bernardo, Silvia Lama, Francesca Miselli, Jenny Bua, Eloisa Gitto, Simona Pesce, Eugenio Baraldi, Daniele Trevisanuto

**Affiliations:** 1 Independent statistician, Solagna, Italy; 2 Department of Women’s and Children’s Health, Neonatal Intensive Care Unit, University Hospital of Padova, Padova, Italy; 3 Neonatal Intensive Care Unit, Children’s Hospital, Azienda Socio Sanitaria Territoriale Spedali Civili, Brescia, Italy; 4 Neonatal Intensive Care Unit, Dipartimento Materno Infantile, Santa Maria della Misericordia Hospital, Perugia, Italy; 5 Critical Care Department, Ospedale Regionale Ca Foncello, Treviso, Italy; 6 L’Unità Operativa Complessa Neonatologia e Terapia Intensiva Neonatale, Dipartimento Materno-Infantile, Azienda Ospedaliera di Rilievo Nazionale Moscati, Avellino, Italy; 7 Department of Pediatrics, Fondazione Poliambulanza, Brescia, Italy; 8 Neonatal Intensive Care Unit, San Bortolo Hospital, Vicenza, Italy; 9 Neonatal Intensive Care Unit, Sant’Anna Hospital, Città della Salute e della Scienza, Torino, Italy; 10 Neonatal Intensive Care Unit, Department of Woman and Child Health and Public Health, Fondazione Policlinico Universitario A. Gemelli Istituto di Ricovero e Cura a Carattere Scientifico, Rome, Italy; 11 Department of Pediatrics, Ospedale dei Bambini V. Buzzi, Milano, Italy; 12 Neonatology and Neonatal Intensive Care Unit, Department of Interdisciplinary Medicine, Aldo Moro University of Bari, Bari, Italy; 13 Neonatologia e Terapia Intensiva Neonatale, Grande Ospedale Metropolitano, Reggio Calabria, Italy; 14 Department of Pediatrics, Neonatal Intensive Care Unit, University Hospital of Verona, Verona, Italy; 15 Division of Neonatology–Neonatal Intensive Care Unit, Hospital of Bolzano, Teaching Hospital of Paracelsus Medical University, Bolzano, Italy; 16 Department of Neuroscience, Psychology, Drug Research and Child Health, Careggi University Hospital, University of Florence, Firenze, Italy; 17 Division of Neonatology, University Hospital of Udine, Udine, Italy; 18 Department of Woman and Child, Buon Consiglio Fatebenefratelli Hospital, Naples, Italy; 19 Neonatal Intensive Care Unit, Department of Woman, Child and Newborn, Fondazione Istituto di Ricovero e Cura a Carattere Scientifico Ca’ Granda Ospedale Maggiore Policlinico, Milan, Italy; 20 Neonatal Intensive Care Unit, Policlinico University Hospital, Modena, Italy; 21 Neonatal Intensive Care Unit, Institute for Maternal and Child Health IRCSS Burlo Garofolo, Trieste, Italy; 22 Department of Pediatrics, University of Messina, Neonatal Intensive Care Unit, Messina, Italy; 23 Maternal and Pediatric Department, San Carlo Hospital, Azienda Ospedaliera Regionale, Potenza, Italy; 24 Department of Women's and Children's Health, University of Padova, Padova, Italy

## Abstract

**Importance:**

Despite continuous improvements in neonatal resuscitation and stabilization in the last decades, thermal management immediately after birth remains an unresolved issue in preterm infants.

**Objective:**

To compare 2 strategies of thermal management (plastic wrapping with or without drying) for preventing heat loss at birth in very preterm infants.

**Design, Setting, and Participants:**

This multicenter, unblinded, randomized clinical trial was conducted among very preterm infants (birth weight <1500 g and/or gestational age ≤30 weeks 6 days) at 21 tertiary care hospitals in Italy from February 21, 2023, to July 18, 2024.

**Interventions:**

Eligible neonates were randomly allocated to either drying before plastic wrapping in the delivery room (intervention arm) or plastic wrapping without drying (control arm).

**Main Outcomes and Measures:**

The primary outcome was the proportion of participants with normothermia (36.5-37.5 °C) at admission to the neonatal intensive care unit (NICU). The secondary outcomes included hypothermia (<36.5 °C), moderate to severe hypothermia (<36.0 °C), and hyperthermia (>37.5 °C) at NICU admission, temperature at 1 hour after NICU admission, intraventricular hemorrhage, respiratory distress syndrome, late-onset sepsis, bronchopulmonary dysplasia, and mortality before hospital discharge.

**Results:**

Overall, 354 very preterm infants were randomized (180 [50.8%] female; mean [SD] gestational age, 28.6 [2.5] weeks); all received the allocated intervention and were included in the analysis. Normothermia at NICU admission was achieved in 81 of 177 dried infants (45.8%) and 82 of 177 undried infants (46.3%; risk ratio, 0.99; 95% CI, 0.79-1.24). The mean (SD) neonatal temperature at NICU admission was 36.4 °C (0.8 °C) in dried neonates and 36.5 °C (0.7 °C) in undried neonates (mean difference, −0.1 °C; 95% CI, −0.2 °C to 0.1 °C). In-hospital mortality included 26 of 177 dried neonates (14.7%) and 10 of 177 undried neonates (5.6%) (unadjusted risk ratio, 2.60; 95% CI, 1.29-5.23). The other secondary outcome measures were not different between the 2 arms.

**Conclusions and Relevance:**

In this multicenter randomized clinical trial, drying before plastic wrapping provided no benefit to very preterm infants in maintaining normothermia at NICU admission. Most deaths could be expected due to the compromised profile of the neonates, with no pathophysiological explanation related to the trial interventions. Approximately half of the infants were outside the normal thermal range at NICU admission; hence, thermal management remains a challenge requiring further investigations.

**Trial Registration:**

ClinicalTrials.gov Identifier: NCT05740072

## Key Points

**Question:**

Does drying before plastic wrapping improve thermoregulation of very preterm infants at birth?

**Findings:**

This multicenter randomized clinical trial including 354 very preterm infants found that drying before plastic wrapping provided no benefit in maintaining normothermia at neonatal intensive care unit admission.

**Meaning:**

This finding does not support the introduction of drying before wrapping in the thermal management bundle in very preterm infants at birth.

## Teaser

This randomized clinical trial compares plastic wrapping with or without drying for preventing heat loss at birth in very preterm infants.

## Introduction

The maintenance of thermal homeostasis is a milestone in neonatology.^1,2,3,4^ Although hypothermia at birth has been clearly associated with adverse neonatal outcomes,^5,6,7,8,9^ the incidence of hypothermia in very preterm infants at admission to neonatal intensive care unit (NICU) remains still high.^9^

International guidelines for neonatal resuscitation suggest several interventions to prevent thermal loss at birth in very preterm infants, such as adequate room temperature, use of infant warmers, polyethylene bags or wrap, preheated mattresses, caps, and heated and humidified gases.^10,11^ Nevertheless, a nonnegligible quota of very preterm infants has hypothermia at NICU admission, suggesting that actual standard of care should be supplemented with further interventions.^4,10,11^

While drying is recommended for thermal management of infants with a gestational age greater than 32 weeks, this procedure is not indicated for very preterm infants, who should be put in a plastic wrap immediately at birth without drying.^10,11^ Of note, such an indication is based on studies comparing wrapping without drying vs drying without wrapping,^12,13^ following previous investigations on plastic wrapping that were not conducted in a delivery room environment.^14,15^ However, the potential advantages of combining those interventions (drying and wrapping) were not explored.

To our knowledge, only 1 study^16^ has investigated the role of drying before wrapping in thermal management of preterm infants, and that study found comparable temperatures after birth in infants wrapped after being dried and in those wrapped without drying. No data are currently available for very preterm infants, who are at higher risk of heat loss due to evaporation.^17^ We hypothesized that drying before wrapping could limit heat loss immediately after birth and improve normothermia at NICU admission in very preterm infants. The present study aimed to compare 2 strategies of thermal management (plastic wrapping with or without drying) for preventing heat loss at birth in very preterm infants.

## Methods

### Study Design

This multicenter, unblinded, randomized clinical trial of drying or not drying before plastic wrapping for the thermoregulation of very preterm infants at birth was performed according to the principles of the Declaration of Helsinki.^18^ The ethics committees of the participating centers approved the trial protocol (coordinating center: Comitato Etico per la Sperimentazione Clinica della Provincia di Padova, Padova, Italy), and parents or guardians provided written informed consent. The study followed the Consolidated Standards of Reporting Trials (CONSORT) reporting guideline. The trial protocol and the statistical analysis plan are available in Supplement 1.

### Setting and Participants

The study was conducted at 21 Italian tertiary hospitals. Neonates satisfying the following inclusion criteria were eligible to participate: (1) estimated birth weight less than 1500 g and/or gestational age less than or equal to 30 weeks 6 days, (2) inborn, and (3) parental consent. Neonates with major congenital malformations (such as cardiac disease, congenital diaphragmatic hernia, abdominal wall defects, and neural tube defects) were excluded.

### Randomization

Before starting the trial, a randomization list (with a 1:1 ratio for the intervention or control arms) was prepared according to a computer-generated, randomized sequence for each participating hospital. Infants of multiple pregnancies were randomized as individuals. The randomized allocation was concealed in double-enclosed, opaque, sealed, and sequentially numbered envelopes. In the delivery room or operating room, the next sequential randomization envelope was opened immediately before the birth of an eligible neonate. The assigned procedure was then performed. Contamination between arms was not allowed.

### Procedures

Before starting the trial, the principal investigator of each hospital (and at least 1 local collaborator) attended a meeting where the study protocol and the procedures were accurately presented. The local neonatal resuscitation teams were educated on protocol details, including eligibility criteria, procedures, and outcome measurement.

Written informed consent was obtained by a member of the neonatal team involved in the study from a parent or guardian at maternal admission to the obstetrics department or before delivery. After obtaining parental consent, eligible neonates were randomly allocated to either drying before wrapping in the delivery room (intervention arm) or wrapping without drying (control arm). Axillary maternal temperature was measured by a digital thermometer (C202; Terumo) about 30 minutes before delivery. Room temperature was measured at delivery by using a wall thermometer (RMR262; Oregon Scientific).

Participants in both arms were managed according to the current guidelines for neonatal resuscitation^4^ that include the steps: (1) room temperature 23 °C to 25 °C; (2) delayed cord clamping (>30 seconds) in uncompromised infants; (3) placing the neonate under the radiant infant warmer with power output set at maximum; (4) covering the body with a plastic bag or wrap to the shoulders (with or without drying according to the randomized assignment); (5) covering the head of the neonate with a cap; (6) using prewarmed mattress (optional); and (7) using heated and humidified gases (optional). Participants in the treatment arm were dried with a prewarmed towel before wrapping, while those in the control arm were not dried before wrapping. All other interventions followed current guidelines for neonatal resuscitation^4^ and were decided by the attending clinician.

These interventions were performed by a neonatologist and a nurse in the delivery or operating room. The interventions were similar in vaginal and cesarean deliveries. Each participating center was allowed to use the polyethylene bag or wrap already in use as local standard of care.

At the end of the stabilization and/or resuscitation, each participant was transferred to the NICU in a transport incubator (with the temperature set at 37 °C). In the NICU, the polyethylene bag or wrap was removed when the infant was put in the incubator.

In all participants, axillary temperature was measured at 3 time points with a digital thermometer (C202): (1) at the end of stabilization and/or resuscitation (just before leaving the delivery room); (2) at NICU admission (primary outcome); and (3) 1 hour after NICU admission. All participants were followed up until discharge or death.

### Outcome Measures

The primary outcome measure was the proportion of participants in the normal thermal range (temperature, 36.5-37.5 °C)^19^ at NICU admission. The secondary outcome measures included the proportions of participants with hypothermia (temperature <36.5 °C), moderate to severe hypothermia (temperature <36.0 °C), or hyperthermia (temperature >37.5 °C) at NICU admission; temperature at 1 hour after NICU admission; occurrence of intraventricular hemorrhage (all grades and grades 3-4), respiratory distress syndrome, late-onset sepsis (defined as infection occurring >72 hours), bronchopulmonary dysplasia (defined as oxygen dependency at 36 weeks’ gestational age); and in-hospital mortality. All predefined serious adverse events (hypothermia <35 °C, hyperthermia >39 °C, and unexpected deaths) were recorded.

### Data Collection

Clinical information and maternal temperature at delivery were recorded by a researcher not involved in neonatal care. Neonatal temperature at NICU admission and after 1 hour were measured by clinicians or nurses. All data were recorded in dedicated data forms and stored in password-protected computers. Personal data were stored in a detached document accessible only by the local principal investigator. Local anonymized datasets were shared with the study principal investigator and then merged for the analysis.

### Masking

In the delivery room, health care practitioners and researchers were not masked to treatment allocation because of intervention features. In the NICU, health care practitioners and researchers were masked to treatment allocation. The statistician was masked to treatment allocation.

### Sample Size

Based on literature data,^20^ we hypothesized that the proportion of neonates with normothermia at NICU admission could increase from 40% in the control arm to 55% in the intervention arm. With power of 80% and type I error of 0.05, the minimum sample size was 346 neonates (173 per arm). As the design was a stratified multicenter individually randomized trial, randomization was balanced and stratified on centers, and we expected no loss of power as a result of randomizing neonates by center.^21^

### Statistical Analysis

All analyses and calculations were performed using R software, version 4.1 (R Foundation for Statistical Computing),^22^ according to the intention-to-treat approach. Statistical significance was set at 5%. An interim analysis was performed in the first 100 participants, with stopping criteria including a statistically significant difference according to Haybittle-Peto boundary^23^ in the primary outcome or a supposed causal association between the intervention and serious adverse events. In the unadjusted analysis, outcome measures were compared between arms using the χ^2^ test, Fisher test, or *t* test. The adjusted analysis used mixed-effect models, including trial arms (fixed effect) and participating center (random effect). Effect sizes were reported as risk ratio (RR) or mean difference with 95% CI. Full details are described in the eMethods in Supplement 2.

## Results

### Interim Analysis

The interim analysis of the first 100 participants did not give any indications for stopping for harm. Normothermia (36.5-37.5 °C) at NICU admission was attained in 25 of 50 dried neonates (50.0%) and 27 of 50 undried neonates (54.0%) (RR, 0.93; 95% CI, 0.63-1.35; *P* = .84). Only 1 case of severe hypothermia (<35 °C) occurred (among dried neonates), while unexpected deaths or severe hyperthermia (>39 °C) did not occur.

### Participant Characteristics

From February 21, 2023, to July 18, 2024, a total of 458 neonates were screened for eligibility. Of these, 104 neonates were excluded because the parents could not be approached for consent (n = 83), the neonate had congenital malformations (n = 10), the parents refused to participate (n = 9), or other reasons (n = 2). Finally, 354 neonates (180 [50.8%] female and 174 [49.2%] male; mean [SD] gestational age, 28.6 [2.5] weeks) were enrolled and randomized to the intervention arm (drying before plastic wrapping [n = 177]) or the control arm (not drying before plastic wrapping [n = 177]). All participants received the allocated intervention. No neonates were lost to follow-up. All randomized infants were included in the analysis (Figure). The arms were balanced with respect to baseline characteristics (Table 1).

**Figure.**
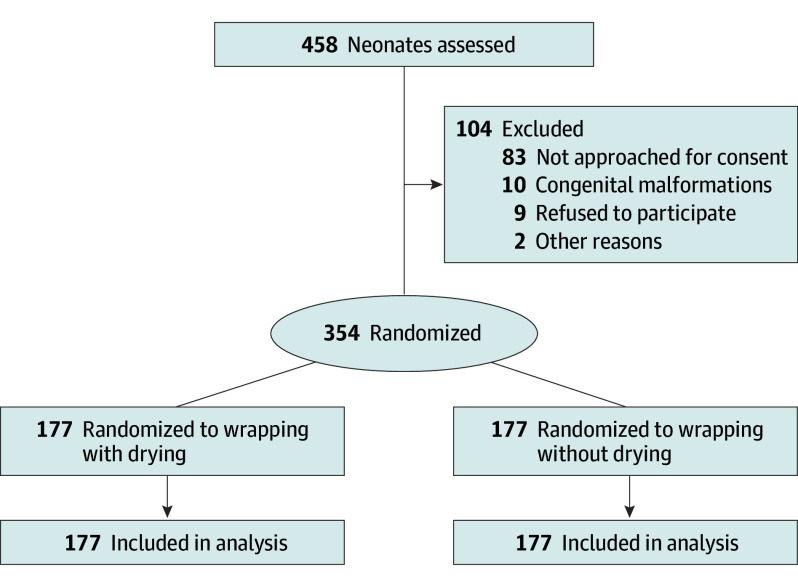
Study Flow Diagram

**Table 1.** Participant Characteristics

Characteristic	Neonate group, No./total No. (%)	
	Drying before plastic wrapping	Not drying before plastic wrapping
**Maternal**		
Age, mean (SD), y^a^	34.3 (6.5)	33.1 (5.1)
Primiparous	71/175 (40.6)	69/176 (39.2)
Antenatal corticosteroid use		
No	13/175 (7.4)	9/176 (5.1)
Incomplete cycle	15/175 (8.6)	21/176 (11.9)
Complete cycle	147/175 (84.0)	146/176 (83.0)
Gestational hypertension	42/177 (23.7)	42/177 (23.7)
Prolonged rupture of the membranes	27/177 (15.3)	19/177 (10.7)
Intrauterine growth restriction	35/177 (19.8)	37/177 (20.9)
Multiple births	48/177 (27.1)	40/177 (22.6)
Maternal temperature at delivery, mean (SD), °C^b^	36.5 (0.6)	36.4 (0.6)
Cesarean delivery	141/177 (79.7)	144/177 (81.4)
Temperature of delivery or operating room, mean (SD), °C^c^	24.2 (1.9)	24.2 (1.9)
**Infant **		
Gestational age, mean (SD), wk	28.8 (2.8)	28.5 (2.4)
Birth weight, mean (SD), g	1086 (342)	1097 (307)
Sex		
Female	91/177 (51.4)	89/177 (50.3)
Male	86/177 (48.6)	88/177 (49.7)
5-min Apgar score, mean (SD)	8.0 (1.3)	8.0 (1.2)
Umbilical cord management		
Immediate cord clamping	116/175 (66.3)	116/172 (67.4)
Delayed cord clamping	59/175 (33.7)	56/172 (32.6)
Thermal interventions		
Infant warmer	177/177 (100)	177/177 (100)
Plastic bag or wrap	177/177 (100)	177/177 (100)
Hat	171/177 (96.6)	167/177 (94.4)
Transwarmer mattress	52/177 (29.4)	50/177 (28.2)
Heated humidified gases	56/177 (31.6)	55/177 (31.1)
Neonatal temperature before transfer to NICU, mean (SD), °C	36.5 (0.6)	36.5 (0.6)
Drugs		
Caffeine	7/177 (4.0)	8/177 (4.5)
Surfactant	15/177 (8.5)	10/177 (5.6)
Maximum respiratory support from delivery room to NICU		
Spontaneous breathing	4/177 (2.3)	7/177 (4.0)
CPAP	121/177 (68.4)	121/177 (68.4)
Intubation	52 (29.4)	49/177 (27.7)
pH, mean (SD)^d^	7.3 (0.1)	7.3 (0.1)
Pco_2_, mean (SD), mm Hg^d^	48.7 (12.2)	48.1 (11.6)
Po_2_, mean (SD), mm Hg^e^	55.9 (20.5)	53.7 (19.1)
Bicarbonate level, mean (SD), mEq/L^f^	21.6 (3.3)	21.7 (2.8)
Absolute base excess, mean (SD), mmol/L^f^	−4.1 (4.3)	−4.0 (3.5)
Lactate level, mean (SD), mg/dL^a^	34.2 (79.3)	27.0 (19.8)
Glycose level, mg/dL^g^	56.5 (32.0)	56.6 (29.3)
Age at NICU admission, mean (SD), min	24 (13)	24 (12)

Abbreviations: CPAP, continuous positive air pressure; NICU, neonatal intensive care unit; Pco_2_, partial pressure of arterial carbon dioxide.

SI conversion factors: To convert bicarbonate to mmol/L, multiply by 1; glucose to mmol/L, multiply by 0.0555; lactate to mmol/L, multiply by 0.111.

^a^ Data were not available for 6 participants.

^b^ Data were not available for 20 participants.

^c^ Data were not available for 7 participants.

^d^ Data were not available for 1 participants.

^e^ Data were not available for 15 participants.

^f^ Data were not available for 2 participants.

^g^ Data were not available for 16 participants.

### Primary Outcome

At NICU admission, normothermia was attained in 81 of 177 dried neonates (45.8%) and 82 of 177 undried neonates (46.3%). The proportion of neonates with normothermia was not statistically different between arms in unadjusted (RR, 0.99; 95% CI, 0.79-1.24) and adjusted (RR, 1.01; 95% CI, 0.74-1.37) analyses (Table 2). Overall, the mean (SD) neonatal temperature at NICU admission was 36.4 °C (0.8 °C) in dried neonates and 36.5 °C (0.7°C) in undried neonates (mean difference, −0.1 °C; 95% CI, −0.2 to 0.1 °C).

**Table 2.** Outcome Measures

Outcome measure	Neonate group, No./total No. (%)		Unadjusted analysis		Analysis adjusted for center	
	Drying before plastic wrapping	Not drying before plastic wrapping	RR (95% CI)	*P* value	RR (95% CI)	*P* value
**Primary**						
Normothermia (36.5-37.5 °C)	81/177 (45.8)	82/177 (46.3)	0.99 (0.79 to 1.24)	.99	1.01 (0.74 to 1.37)	.97
**Secondary**						
Hypothermia (<36.5 °C)	89/177 (50.3)	81/177 (45.8)	1.10 (0.88 to 0.36)	.46	1.18 (0.88 to 1.60)	.27
Moderate to severe hypothermia (<36.0 °C)	40/177 (22.6)	34/177 (19.2)	1.18 (0.78 to 1.77)	.51	1.27 (0.81 to 2.01)	.30
Hyperthermia (>37.5 °C)	7/177 (4.0)	14/177 (7.9)	0.50 (0.21 to 1.21)	.18	0.47 (0.19 to 1.17)	.10
Temperature after 1 h, mean (SD), °C^a^	36.3 (0.8)	36.4 (0.6)	−0.1 (−0.3 to 0.1)	.20	−0.1 (−0.2 to 0.1)	.12
IVH (all grades)	30/175 (17.1)	35/177 (19.8)	0.87 (0.56 to 1.35)	.62	0.86 (0.53 to 1.39)	.53
IVH (grades 3-4)	14/175 (8.0)	14/177 (7.9)	1.01 (0.50 to 2.06)	.99	1.01 (0.48 to 2.08)	.98
RDS	137/176 (77.8)	139/177 (78.5)	0.99 (0.89 to 1.11)	.98	0.97 (0.77 to 1.23)	.81
Late-onset sepsis	32/176 (18.2)	38/177 (21.5)	0.85 (0.56 to 1.29)	.52	0.84 (0.52 to 1.34)	.45
BPD	44/167 (26.3)	52/174 (29.9)	0.88 (0.63 to 1.24)	.54	0.84 (0.56 to 1.26)	.40
Mortality	26/177 (14.7)	10/177 (5.6)	2.60 (1.29 to 5.23)	.008	2.71 (1.31 to 5.62)	.007

Abbreviations: BPD, bronchopulmonary dysplasia; IVH, intraventricular hemorrhage; RDS, respiratory distress syndrome; RR, risk ratio.

^a^ Adjusted and unadjusted analyses are reported as the mean difference (95% CI).

### Secondary Outcomes

There was no evidence that the secondary outcomes differed between arms, apart from mortality (Table 2). In-hospital mortality included 26 of 177 (14.7%) in dried neonates and 10 of 177 (5.6%) in undried neonates (unadjusted RR, 2.60 [95% CI, 1.29-5.23]; adjusted RR, 2.71 [95% CI, 1.31-5.62]) (Table 2). Post hoc analyses confirmed that the drying arm was associated with increased mortality after adjusting for clinically relevant confounders (sex, gestational age, multiple births, intrauterine growth restriction, intubation in the delivery room, temperature at NICU admission) (RR, 3.08; 95% CI, 1.44-6.56) or preintervention clinically relevant confounders (sex, gestational age, multiple births, intrauterine growth restriction) (RR, 3.14; 95% CI, 1.50-6.58) (eTable 1 in Supplement 2). The description of deceased neonates is reported in eTables 2 and 3 in the Supplement 2.

### Safety

Few cases of severe hypothermia (temperature <35 °C) occurred, without statistically significant difference between arms (Table 3). There were no unexpected deaths or severe hyperthermia (temperature >39 °C).

**Table 3.** Serious Adverse Events

Outcome measure	Neonate group, No. (%)		Unadjusted analysis	
	Drying before plastic wrapping (n = 177)	Not drying before plastic wrapping (n = 177)	RR (95% CI)	*P* value
Unexpected deaths	0	0	NA	NA
Severe hypothermia (temperature <35 °C)	7 (4.0)	3 (1.7)	2.33 (0.61-8.88)	.33
Severe hyperthermia (temperature >39 °C)	0	0	NA	NA

Abbreviations: NA, not applicable; RR, risk ratio.

### Subanalysis Stratified by Gestational Age

In 102 participants with gestational age between 23 weeks 0 days and 27 weeks 6 days, the drying arm had a lower neonatal temperature at 1 hour (mean difference, −0.3 °C; 95% CI, −0.6 to −0.1 °C) and increased mortality (RR, 4.71; 95% CI, 2.00-11.12) (eTable 4 in Supplement 2). In 213 participants with gestational age between 28 weeks 0 days and 31 weeks 6 days, the outcomes were not statistically different between arms (eTable 5 in Supplement 2). The remaining 39 participants had gestational age between 32 weeks 0 days and 35 weeks 6 days but satisfied the inclusion criteria regarding birth weight.

### Sensitivity Analysis of Binary Outcomes

Adjusted analysis of binary outcomes using modified Poisson models with robust SEs generally confirmed the findings of the main analysis, subanalyses, and post hoc analysis of mortality (eTables 6-9 in Supplement 2). Moreover, this sensitivity analysis suggested lower proportion of hyperthermia in the drying arm (RR, 0.50; 95% CI, 0.26-0.95) (eTable 6 in Supplement 2).

### Sensitivity Analysis With Adjustment for Multiple Testing

Sensitivity analyses with Benjamini-Hochberg adjustment for multiple testing confirmed the association between the drying arm and increased mortality in neonates with gestational age between 23 weeks 0 days and 27 weeks 6 days (RR, 4.71; 95% CI, 1.08-20.76; adjusted *P* = .03) (eTable 10 in Supplement 2). This sensitivity analysis yielded statistically nonsignificant results for all other comparisons between arms (eTable 10 in Supplement 2).

## Discussion

In this multicenter randomized clinical trial, drying before plastic wrapping provided no benefit to very preterm infants in maintaining normothermia at NICU admission. There were few adverse events, but a higher mortality rate was observed in neonates who were dried before plastic wrapping.

Despite continuous improvements in neonatal resuscitation and stabilization in the last decades, thermal management immediately after birth remains an unresolved issue in preterm infants.^3,16,24^ The Vermont Oxford Network reported that hypothermia in very-low-birth-weight infants at NICU admission had been reduced from 52.6% to 38.2% during a 7-year period, but approximately 4 in 10 infants were still cold at NICU admission.^25^ In addition, evidence from the literature underlines the importance of avoiding both hyperthermia and hypothermia after birth.^7,8,10,13^ The International Liaison Committee on Resuscitation (ILCOR) Neonatal Life Support Task Force confirmed the recommendation about using combined interventions for maintaining normothermia in preterm infants immediately after birth.^26^

To accomplish such a goal, the present trial investigated the opportunity of extending to very preterm infants a component of the thermal intervention (drying), which is currently recommended for infants with gestational age greater than 32 weeks.^10,11^ While it is recognized that wrapping acts as a barrier against heat loss due to evaporation,^2,17^ we hypothesized that drying the infant could improve this thermal control by reducing the mechanism of evaporation. However, our findings did not support such a hypothesis, as normothermia and neonatal temperature at NICU admission were comparable between very preterm infants who did or did not undergo drying. This result confirmed the conclusions of a previous small study that was conducted in larger infants (28-37 weeks’ gestation).^16^ Hence, we can speculate that (1) drying delays wrapping and may increase thermal loss during this time interval, and/or (2) drying before wrapping may not impact the mechanism of evaporation compared with immediate wrapping. Nonetheless, drying a neonate before placing him or her in a polyethylene bag takes time, and this happens at a crucial time when thermoregulation and resuscitation of the neonate are occurring in parallel in a busy delivery room. Our findings suggest that drying should not be performed prior to placing the neonate in a polyethylene bag.

Previous studies associated deviations from normothermia at NICU admission with increased likelihood of mortality and morbidity, highlighting the importance of avoiding both hyperthermia and hypothermia after birth.^7,8^ In our study, most clinical secondary outcomes were comparable between the 2 arms, as drying before wrapping and immediate wrapping led to comparable temperatures at NICU admission. Interestingly, hyperthermia seemed more frequent in undried infants, but this was confirmed in only 1 analytical approach, hence suggesting caution in the interpretation. However, the mortality rate was surprisingly higher among dried neonates, which was unexpected given the safety of the procedures and the comparable temperatures at NICU admission between the study arms. This finding was consistent in more than 1 sensitivity analysis and required further attention. We reviewed the clinical records of each deceased neonate to shed light on this finding, and we observed that most deaths could be expected due to the compromised profile of the neonate (extremely preterm infants and/or disease severity). Moreover, the analysis stratified by gestational age suggested that the subgroup of smaller infants (gestational age between 23 weeks 0 days and 27 weeks 6 days) contributed to the mortality difference between the study arms. On the other hand, we could not exclude that the process of drying the infant immediately after birth could have determined some degree of cardiovascular instability due to the manipulation of the smaller infants. In the end, we could not reasonably find a pathophysiological explanation related to the trial interventions; therefore, we believe that the mortality difference between the study arms could be a random finding. Of note, drying is recommended for the thermal management of infants with gestational age greater than 32 weeks,^10,11^ but our findings suggested that such a procedure was not harmful in infants with gestational age of 28 to 32 weeks. Nonetheless, the importance of this critical outcome cannot be understated, hence further studies are needed to fully disclose such information.

Of note, approximately half of the neonates were outside the normal thermal range at NICU admission. This finding highlights that maintaining normothermia after birth is still an important issue.^7,9,17,20,27,28,29^ International guidelines stressed the importance of implementing a bundle of interventions to prevent thermal loss at birth in preterm infants.^10,11^ We explored the potential benefit of adding drying before wrapping in the thermal bundle, but the findings did not support this intervention. Our data showed an adequate adherence concerning using an infant warmer, using plastic wrapping, and covering the head among participants, while a transwarmer mattress and heated humidified gases were used in only 1 of 3 participants. Increasing the use of the transwarmer mattress and heated humidified gases may improve thermal management after birth, as suggested by recent ILCOR recommendations.^26^ Other components of the thermal bundle such as checklist and debriefing may play an important role in this matter,^17^ but such information was not collected in our trial.

### Strengths and Limitations

To our knowledge, this is the first large trial investigating the role of drying before wrapping in the thermal management of very preterm infants. The strengths of the trial include the multicenter design, the large sample size, and the adherence to the assigned treatment without loss to follow-up.

The study limitations include the unmasking of health care practitioners and researchers in the delivery room and the absence of after-discharge assessments. Furthermore, the generalizability of the findings should be limited to similar settings. Baseline data from our participating centers revealed a high rate of cesarean deliveries, a low rate of delayed cord clamping, and limited use of thermal mattress and heated humified gases, which might have contributed to the high rate of hypothermia at NICU admission.

## Conclusions

In this multicenter randomized clinical trial, drying before plastic wrapping provided no benefit to very preterm infants in maintaining normothermia at NICU admission. Approximately half of the infants were outside the normal thermal range at NICU admission. Most deaths could be expected due to the compromised profile of the neonates, and we could not reasonably find a pathophysiological explanation related to the trial interventions. Thermal management of such vulnerable infants is still a challenge and needs further investigations on suitable improvements of the current thermal intervention bundle.

## Notes

Supplement 1.
Trial Protocol and Statistical Analysis Plan

Supplement 2. **eMethods.** Description of the statistical methods **eTable 1.** Multivariable Analysis of Mortality **eTable 2.** Timing and Cause of Death of Deceased Neonates **eTable 3.** Information at NICU Admission of Deceased Neonates **eTable 4.** Outcome Measures in Neonates With Gestational Age Between 23^+0^ and 27^+6^ Weeks **eTable 5.** Outcome Measures in Neonates With Gestational Age Between 28^+0^ and 31^+6^ Weeks **eTable 6.** Adjusted Analysis of the Binary Outcome Measures by Using a Modified Poisson Model With Robust Standard Errors **eTable 7.** Adjusted Analysis of the Binary Outcome Measures by Using a Modified Poisson Model With Robust Standard Errors in Neonates With Gestational Age Between 23^+0^ and 27^+6^ Weeks **eTable 8.** Adjusted Analysis of the Binary Outcome Measures by Using a Modified Poisson Model With Robust Standard Errors in Neonates With Gestational Age Between 28^+0^ and 31^+6^ Weeks **eTable 9.** Multivariable Analysis of Mortality by Using a Modified Poisson Model With Robust Standard Errors **eTable 10.** Sensitivity Analysis With Adjustment for Multiple Testing Using Benjamini–Hochberg Procedure

Supplement 3.
Nonauthor Collaborators

Supplement 4.
Data Sharing Statement
