# Supplementary material for: Drying Very Preterm Infants Before Plastic Wrapping at Birth: A Randomized Clinical Trial
Source: JAMA Netw Open. 2026 Mar 3;9(3):e2556902. doi: 10.1001/jamanetworkopen.2025.56902 (PMC12958082; doi:10.1001/jamanetworkopen.2025.56902)
Supplement: Supplement 1. — Trial Protocol and Statistical Analysis Plan [file jamanetwopen-e2556902-s001.pdf]

TITLE: Effect of drying before plastic wrapping on thermal losses in very preterm infants at birth: a multicenter, randomized controlled trial

Daniele Trevisanuto, Azienda Ospedaliera Università di Padova, Padova, (coordinator center) (daniele.trevisanuto@unipd.it)

Nicoletta Doglioni, Azienda Ospedaliera Università di Padova, Padova (nicolettadoglioni@yahoo.it)

Benedetta Bua, Azienda Ospedaliera Università di Padova, Padova (benedetta.bua89@gmail.com)

Francesco Cavallin, Independent statistician, Solagna (cescocava@libero.it)

Correspondence to:

Daniele Trevisanuto, MD, Department of Women's and Children's Health, University Hospital of Padua, Via Giustiniani, 3, 35128 Padova, Italy

Phone ++39 049 8213545; Fax ++ 39 049 8213301; e-mail: daniele.trevisanuto@unipd.it

ABSTRACT

Background: Hypothermia in preterm infants during the immediate postnatal phase is associated with morbidity and mortality and remains an unresolved, worldwide challenge.

A list of interventions, including adequate room temperature, use of infant warmers, polyethylene bags/wrap, pre-heated mattresses, caps and heated and humidified gases, to prevent thermal loss at birth in very preterm infants has been recommended, but a certain percentage of very preterm infants are hypothermic at the time of the neonatal intensive care unit (NICU) admission suggesting that further measures are needed. While drying is recommended for the thermal management of infants with gestational age >32 weeks, this procedure is not indicated for very preterm infants who should be put in a plastic wrap immediately at birth without drying. However, such indication is based on studies comparing wrapping without drying vs. drying without wrapping, while the potential advantages of combining these interventions were not explored. We hypothesized that drying before wrapping could prevent heat loss immediately after birth and reduce hypothermia at NICU admission in very preterm infants.

Objective: The aim of this study will be to compare two modes of thermal management (plastic wrapping with or without drying) for preventing heat loss at birth in very preterm infants.

Methods: This is a multicenter, unblinded, randomized controlled trial comparing drying vs. not drying before plastic wrapping for the thermoregulation of very preterm infants at birth. After obtaining parental consent, all infants with estimated birth weight <1500 g and/or gestational age $\leq 30^{+6}$ weeks will be assigned to be managed with or without drying before plastic wrapping. Room temperature and maternal temperature will be measured at the time of delivery. Patients allocated in both groups will be managed based on the current guidelines for neonatal resuscitation. The primary outcome measure will be the proportion of neonates in the normal thermal range (temperature 36.5-37.5°C) at NICU admission. Secondary outcome measures will be: proportion of neonates with hypothermia (<36.5°C and <36.0°C) at NICU admission; proportion of hyperthermic neonates (temperature >37.5°C) at NICU admission; temperature at 1 hour after NICU admission; proportion

of intraventricular hemorrhage; proportion of respiratory distress syndrome; proportion of late onset sepsis; proportion of bronchopulmonary dysplasia; mortality before hospital discharge.

Trial registration: the study will be registered in ClinicalTrials.gov after EC approval.

Keywords: delivery room, hypothermia, preterm infant, drying, plastic wrapping

Funds: this is a no-profit study. No funds have been planned for this study.

Background

The maintenance of thermal homeostasis is a milestone in neonatology (1-4). A significant association between hypothermia at delivery and adverse neonatal outcomes has been clearly demonstrated (5-8), but the incidence of hypothermia in very preterm infants at the time of admission to neonatal intensive care unit (NICU) in Europe remains still high (8).

International guidelines for neonatal resuscitation suggest several interventions to prevent thermal loss at birth in very preterm infants, such as adequate room temperature, use of infant warmers, polyethylene bags/wrap, pre-heated mattresses, caps and heated and humidified gases (9,10). Nevertheless, a certain percentage of very preterm infants are hypothermic at the time of the NICU admission suggesting that, in addition to the actual standard of care, further interventions are needed (4,9,10).

While drying is recommended for the thermal management of infants with gestational age >32 weeks (9,10), this procedure is not indicated for very preterm infants who should be put in a plastic wrap immediately at birth without drying. Of note, such indication is based on studies comparing wrapping without drying vs. drying without wrapping (11,12), following previous investigations on plastic wrapping which were not conducted in the delivery room environment (13,14). However, the potential advantages of combining those interventions (drying and wrapping) were not explored. To our knowledge, only one study investigated the role of drying before wrapping in the thermal management of preterm infants, and found comparable temperatures after birth in infants wrapped after being dried and in those wrapped without drying (15). No data are currently available for very preterm infants, who are at higher risk of heat loss due to evaporation (16). We hypothesized that drying before wrapping could prevent heat loss immediately after birth and reduce hypothermia at NICU admission in very preterm infants.

The "PICOT" question of this study is:

P: in very preterm infants,

I: does drying before plastic wrapping in the delivery room,

C: compared to standard of care (plastic wrapping without drying),

O: increase the percentage of infants in the normal thermal range (temperature 36.5-37.5°C),

T: at the time of admission to the NICU?

The objective of this study will be to compare two modes of thermal management (plastic wrapping with or without drying) for preventing heat loss at birth in very preterm infants.

Methods/Design

Study design

This is a multicenter, unblinded, randomized controlled trial comparing drying vs. not drying before plastic wrapping for the thermoregulation of very preterm infants at birth.

Setting

The study will be conducted at 21 Italian Level III hospitals.

Inclusion criteria

Infants satisfying the following inclusion criteria will be eligible to participate in the study:

1. Estimated birth weight <1500 g and/or gestational age $\leq 30^{+6}$ weeks (and)
2. Inborn (and)
3. Parental consent; a written informed consent will be obtained by a member of the neonatal team involved in the study from a parent or guardian at the maternal admission to the Obstetric Department.

Exclusion criteria

1. Major congenital malformations (i.e. cardiac disease, defects of abdominal wall, ...);
2. Outborn;
3. Parental refusal to participate to the study.

Procedure

Written and oral information will be offered to parents by the attending physician at maternal admission to the obstetrical ward or before delivery. After obtaining parental consent, all infants with estimated birth weight <1500 g and/or gestational age $\leq 30^{+6}$ weeks will be assigned to be managed with or without drying before plastic wrapping in the delivery room immediately after birth. Axillary maternal temperature will be measured by a digital thermometer (C202; Terumo,

Tokyo, Japan) about 30 minutes before delivery. Room temperature will be measured at the time of delivery by using a wall thermometer (Oregon Scientific RMR262) in all the study sites.

Patients allocated in both arms will be managed based on the current guidelines for neonatal resuscitation that include the following steps:

- Maintain room temperature at least 24°C;
- Delayed cord clamping (>30 seconds) in uncompromised infants;
- Place the neonate under the radiant infant warmer with the power output set at maximum;
- Cover the body with a plastic bag/wrap up to the shoulders (with or without drying according to the randomized assignment);
- Cover the head of the baby with a cap;
- Use a pre-warmed mattress (optional);
- Use heated and humidified gases (optional).

In case of assignment to the treatment arm, the infant will be dried with a pre-warmed towel before plastic wrapping. In case of assignment to the control arm, the infant will not be dried before plastic wrapping.

All the other interventions, including CPAP, mechanical ventilation, supplemental oxygen concentrations, administration of chest compressions and/or medications will follow the current guidelines for neonatal resuscitation and the decision will be taken by the neonatal team.

At the end of the stabilization, the patient will be transferred to the NICU in a transport incubator (with the temperature set at 37°C).

In all participants, axillary temperature will be measured at 3 time points with a digital thermometer (C202; Terumo, Tokyo, Japan): a) at the end of the stabilization (before leaving the delivery room); b) at NICU admission (primary outcome); and c) 1 hour after NICU admission. Duration of the study will be until the patient's discharge or death.

Primary outcome measure

1. The primary outcome measure will be the proportion of neonates in the normal thermal range (temperature 36.5-37.5°C) at NICU admission.

Secondary outcome measures

2. Proportion of neonates with hypothermia (temperature <36.5°C) at NICU admission;
3. Proportion of neonates with moderate-severe hypothermia (temperature <36.0°C) at NICU admission;
4. Proportion of hyperthermic neonates (temperature >37.5°C) at NICU admission;
5. Temperature at 1 hour after NICU admission;
6. Proportion of intraventricular hemorrhage (all grades and grade III-IV);
7. Proportion of respiratory distress syndrome (RDS);
8. Proportion of late onset sepsis;
9. Proportion of bronchopulmonary dysplasia;
10. Mortality before hospital discharge.

Generalizability

The findings of this study will be important for other level III hospitals where the majority of high risk pregnancies are referred, but also for level I and II centers where, sometimes preterm infants are born. The generalizability of the study is based on the large number of participating centers, representing different realities and different approaches to the delivery room management of very preterm infants. Based on the results of the present study, we could speculate whether drying the infant at birth may have a role in increasing the proportion of very preterm in the normal thermal range at NICU admission. Furthermore, potential complications, such as hyperthermia will be strictly monitored and collected.

Sample size

Based literature data (17), we hypothesize that the proportion of neonates in the normal thermal range at NICU admission could increase from 40% in the control arm to 55% in the intervention arm. With a power of 80% and an error I type of 0.05, the minimum sample size is estimated in 346 neonates (173 per arm). As the design is a stratified multicenter individually randomized trial, randomization is balanced and stratified on centers, and we expect no loss of power as a result of randomizing neonates by center (18). Sample size calculations were performed using R 4.1 software (R Foundation for Statistical Computing, Vienna, Austria) (19).

Recruitment

Written and oral information will be offered to parents at maternal admission to the Obstetric Department by the attending physician. Informed written consent will be signed by a parent. A senior investigator will be available at all times to discuss concerns raised by parents or clinicians during the course of the trial.

Randomization

Eligible infants will be randomly assigned to intervention or control arms in a 1:1 ratio according to a computer-generated, randomized sequence for each participating hospital. Infants of multiple pregnancies will be randomized as individuals. Randomization (with blocks of size 4-6) will be stratified by center. The randomized allocation will be concealed in double-enclosed, opaque, sealed, and sequentially numbered envelopes prepared at University Hospital of Padua. In the delivery room/operating room, the next sequential randomization envelope will be opened only when the neonate will be considered to be eligible by the attending operator. The assigned procedure will be then performed. Contamination between arms will not be allowed.

Blinding

Due to the characteristics of the intervention, neither caregivers nor outcome assessors will be masked to treatment allocation. However, the statistician who will perform data analysis will be blind for treatment allocation. To minimize bias, strict criteria and definitions will be maintained during the trial.

Guidelines for Management

Before starting the study, the PI of each hospital (and at least one local collaborator) involved in the study will participate to a meeting where all the details of the study protocol will be presented. The local neonatal resuscitation teams responsible of the delivery room management will be educated on the details of the protocol including temperature measurements in term of correct modality (axillary), time of the study, collection of the data.

Data collection

Data will be recorded in a data sheet designed for this study. All data will be collected by an observer not involved in the care of the neonates. The following clinical information will be registered: eligibility, antenatal history, randomization, all data above listed in 'Primary outcome measure', 'Secondary outcome measures' sections. Further information will be collected on expected serious adverse events (SAEs). Personal information about potential and enrolled participants will be collected, shared, and maintained in order to protect confidentiality before, during, and after the trial by the local P.I. in a personal PC protected by password. The P.I. and the members of the steering committee located in Padua will have access to the final trial dataset.

Statistical analysis

Analysis will be performed according to the "intention to treat" approach. In case of deviations from the protocol regarding the contamination between arms, a per-protocol analysis will be also performed and the conclusion will be drawn according to the results of both analyses. Continuous

data will be expressed as mean and standard deviation, or median and interquartile range. Outcome measures will be compared between the two arms using Chi-square test, Fisher's test, Student *t* test or Mann-Whitney test as appropriate (unadjusted analysis). Regression models (adjusted analysis) will be estimated to evaluate the effect of drying on the outcome measures, adjusting for participating centers and potential confounders (i.e. clinically relevant variables or unbalanced characteristics at baseline). Effect sizes will be reported as risk ratio or mean difference with 95% confidence intervals. A sub-analysis according to gestational age (23^{+0}-27^{+6} and 28^{+0}-31^{+6} weeks) will be performed with exploratory purpose. A p-value less than 0.05 will be considered statistically significant. Statistical analysis will be performed using R 4.1 software (R Foundation for Statistical Computing, Vienna, Austria) (19).

Ethical considerations

Written parental consent is necessary before enrollment of the patients in the study. We consider that there will be not risks for both study groups. Hypothermia as well as hyperthermia episodes will be strictly monitored in both groups.

Ethics Committee approval

The study will be approved by the Ethics Committees for Human Investigation of the participating hospitals.

Compliance to protocol

Compliance will be defined as full adherence to protocol. Compliance with the protocol will be ensured by the local PI and collaborators in each hospital; they will be responsible for local data collection. The local PI will weekly monitor the adherence to the study protocol and will input the patients' data in an electronic data sheet. Double data entry will be performed by 2 independent members at each center to promote data quality.

Missing data

Investigators and study staff will be trained on the importance of the completion of the study period of enrolled patients. Parents will also be informed about this crucial aspect to reduce dropout, and a local investigator will be available at each site anytime the parents may need further information or clarification during the study period.

Data Safety and Monitoring Board

Safety measures will include incidence, severity and causality of reported SAEs, represented by changes in occurrence of the expected common neonatal complications and the development of unexpected SAEs. SAE will be defined as unexpected death, severe hyperthermia (temperature >39°C) and hypothermia (temperature <35°C). All SAEs will be followed until complete resolution or until the clinician responsible for the care of the recruited patient considers the event to be chronic or the infant to be stable.

A monitoring board including an independent assessor (not involved in the study) from the Azienda Ospedale Università di Padova and assessors from each participating hospital will review all the deaths and adverse effects. If there is a reasonable suspected causal relationship with the intervention, SAEs will be reported to the Ethics Committee to guarantee the safety of the participants.

An interim analysis will be performed on the primary endpoint and SAEs from the first 100 infants enrolled. The interim analysis will be performed by the statistician, blinded for the treatment allocation, who will report to the principal investigator. The principal investigator will discuss the results of the interim analysis with the monitoring board and the trial will be ended in case of harm. Criteria for stopping for harm include: a statistically significant difference in the primary outcome between the treatment groups; a reasonable suspected causal relationship between the intervention and SAEs.

Confidentiality

Only local P.I. will have access to the electronic database with an assigned personal account and password. Subjects will be identified by sex, birth date, and assigned trial number, during and after the trial, in accordance with personal data protection law.

Access to data

The principal investigator of each site will have complete access to the final trial dataset, and no contractual agreement exists to limit such access for the investigators.

Dissemination policy

The results of the trial are expected to be published in a scientific journal and to be presented in medical seminars and conferences. The final reporting will follow the CONSORT Report guidelines (http://www.consort-statement.org).

Discussion

There are unique features of this trial compared to prior studies on prevention of heat loss at birth in very preterm infants. International guidelines for neonatal resuscitation recommend putting very preterm infants in a plastic wrap immediately at birth without drying (9,10), but the potential advantages of combining those interventions (drying and wrapping) are not considered. In this trial, we will compare two modalities of thermal management (plastic wrapping with or without drying) for preventing heat loss at birth in very preterm infants. The results of this study will allow to cover this gap of knowledge and, possibly, will help to improve thermal management of very preterm infants at birth that remains an unresolved matter in high and low-resource settings.

Trial status

The trial is being submitted to the Ethics Committees of the coordinator center and, subsequently, to the Ethics Committees of the participating hospitals.

Competing interests

The authors declare that they have no competing interests.

Authors' contributions

All authors have made substantial contributions to the conception and design of the study protocol and have given final approval of the actual version. FC has prepared the statistical design, including the sample size calculation, and has given the approval of the actual version. All authors read and approved the actual version of the protocol.

References

1. Sedin G. To avoid heat loss in very preterm infants. J Pediatr 2004;145:720-2.
2. Trevisanuto D, Sedin G. Thermal homeostasis and clinical management. In: Neonatology. A Practical Approach to Neonatal Diseases. Buonocore G, Bracci R, Weindling M. Springer Ed 2016.
3. Lunze K, Bloom DE, Jamison DT, Hamer DH. The global burden of neonatal hypothermia: systematic review of a major challenge for newborn survival. BMC Med. 2013;11:24.
4. Perlman JM, Wyllie J, Kattwinkel J, Wyckoff MH, Aziz K, Guinsburg R, Kim HS, Liley HG, Mildenhall L, Simon WM, Szyld E, Tamura M, Velaphi S; Neonatal Resuscitation Chapter Collaborators. Part 7: Neonatal Resuscitation: 2015 International Consensus on Cardiopulmonary Resuscitation and Emergency Cardiovascular Care Science With Treatment Recommendations. Circulation. 2015;132(16 Suppl 1):S204-41.

5. Miller SS, Lee HC, Gould JB. Hypothermia in very low birth weight infants: distribution, risk factors and outcomes. J Perinatol 2011;31 (Suppl 1):S49-56.
6. Laptook AR, Salhab W, Bhaskar B, Neonatal Research Network. Neonatal Research Network. Admission temperature of low birth weight infants: predictors and associated morbidities. Pediatrics 2007; 119:e643-649.
7. Lyu Y, Shah PS, Ye XY, Warre R, Piedboeuf B, Deshpandey A, Dunn M, Lee SK; Canadian Neonatal Network. Association between admission temperature and mortality and major morbidity in preterm infants born at fewer than 33 weeks' gestation. JAMA Pediatr. 2015;169(4):e150277.
8. Wilson E, Maier RF, Norman M, Misselwitz B, Howell EA, Zeitlin J, Bonamy AK; Effective Perinatal Intensive Care in Europe (EPICE) Research Group. Admission Hypothermia in Very Preterm Infants and Neonatal Mortality and Morbidity. J Pediatr. 2016;175:61-67.e4.
9. Wyckoff MH, Aziz K, Escobedo MB, Kapadia VS, Kattwinkel J, Perlman JM, Simon WM, Weiner GM, ZaichKin JG. Part 13: Neonatal Resuscitation: 2015 AHA Guidelines Update for CardiopulmonaryResuscitation and Emergency Cardiovascular Care. Circulation 2015; 132 (suppl 2):S543-S560.
10. Wyllie J, Bruinenberg J, Roehr CC, Rüdiger M, Trevisanuto D, Urlesberger B. European Resuscitation Council Guidelines for Resuscitation 2015: Section 7. Resuscitation and support of transition of babies at birth. Resuscitation. 2015;95:249-63.
11. Vohra S, Frent G, Campbell V, Abbott M, Whyte R. Effect of polyethylene occlusive skin wrapping on heat loss in very low birth weight infants at delivery: a randomized trial. J Pediatr. 1999 May;134(5):547-51.
12. McCall EM, Alderdice F, Halliday HL, Vohra S, Johnston L. Interventions to prevent hypothermia at birth in preterm and/or low birth weight infants. Cochrane Database Syst Rev. 2018;2(2):CD004210

13. Baumgart S, Engle WD, Fox WW, Polin RA. Effect of heat shielding on convective and evaporative heat losses and on radiant heat transfer in the premature infant. J Pediatr 1981;99:948-56
14. Baumgart S. Reduction of oxygen consumption, insensible water loss, and radiant heat demand with use of a plastic blanket for low-birth-weight infants under radiant warmers. Pediatrics 1984;74:1022-8.
15. Cardona Torres LM, Amador Licona N, Garcia Campos ML, Guizar-Mendoza JM. Polyethylene wrap for thermoregulation in the preterm infant: a randomized trial. Indian Pediatr. 2012 Feb;49(2):129-32.
16. Trevisanuto D, Testoni D, de Almeida MFB. Maintaining normothermia: Why and how? Semin Fetal Neonatal Med. 2018;23(5):333-339.
17. Cavallin F, Doglioni N, Allodi A, Battajon N, Vedovato S, Capasso L, Gitto E, Laforgia N, Paviotti G, Capretti MG, Gizzi C, Villani PE, Biban P, Pratesi S, Lista G, Ciralli F, Soffiati M, Staffler A, Baraldi E, Trevisanuto D; Servo COntrol for PReterm Infants (SCOPRI) Trial Group. Thermal management with and without servo-controlled system in preterm infants immediately after birth: a multicentre, randomised controlled study. Arch Dis Child Fetal Neonatal Ed. 2021 Nov;106(6):572-577.
18. Vierron E, Giraudeau B. Design effect in multicenter studies: gain or loss of power? BMC Med Res Methodol 2009;9:39.
19. R Core Team (2022). R: A language and environment for statistical computing. R Foundation for Statistical Computing, Vienna, Austria. URL https://www.R-project.org/.

Statistical Analysis Plan

TRIAL FULL TITLE	Effect of drying before plastic wrapping on thermal losses in very preterm infants at birth: a multicenter, randomized controlled trial
ACRONYM	NEODRY Trial
CLINICALTRIALS.GOV	ClinicalTrials.gov NCT05740072
PROTOCOL VERSION	Version 1.1, 19 Dec 2022
SAP VERSION	Version 1.0, 19 Dec 2022
TRIAL STATISTICIAN	Francesco Cavallin
TRIAL CHIEF INVESTIGATOR	Daniele Trevisanuto
SAP AUTHORS	Francesco Cavallin, Daniele Trevisanuto

Table of Contents

1 Abbreviations and Definitions 3

2 Introduction 4

2.1 Summary 4

2.2 Purpose of the analyses 4

3 Study Objectives and Endpoints 4

3.1 Study Objectives 4

3.2 Endpoints 4

4 Study Methods 5

4.1 General Study Design and Plan 5

4.2 Inclusion-Exclusion Criteria and General Study Population 5

4.3 Randomization and Blinding 5

4.4 Study Variables 6

5 Sample Size 6

6 General Considerations 6

6.1 Timing of Analyses 6

6.2 Approach for main analysis 6

6.3 Additional analysis 6

6.4 Missing Data 6

6.5 Interim Analyses and Data Monitoring 7

7 Summary of Study Data 7

8 Analysis of primary outcome measure 7

9 Analysis of secondary outcome measures 7

10. Exploratory Analyses 8

10 Safety Analyses 8

11 Other Analyses 8

13 Summary of Changes to the Protocol 8

14 References 8

1 Abbreviations and Definitions

ITT	Intention-to-treat
NICU	Neonatal Intensive Care Unit
SAE	Serious adverse event
SAP	Statistical Analysis Plan

2 Introduction

2.1 Summary

The maintenance of thermal homeostasis is a milestone in neonatology (1). Hypothermia at delivery has been clearly associated with adverse neonatal outcomes, but the incidence of hypothermia in very preterm infants at the time of admission to neonatal intensive care unit (NICU) in Europe remains still high (2). Although international guidelines for neonatal resuscitation suggest several interventions to prevent thermal loss at birth in very preterm infants (such as adequate room temperature, use of infant warmers, polyethylene bags/wrap, pre-heated mattresses, caps and heated and humidified gases), a proportion of very preterm infants are hypothermic at NICU admission, hence suggesting the need for further interventions (3,4). Drying is recommended for the thermal management of infants with gestational age >32 weeks (3,4), but this procedure is not indicated for very preterm infants who should be put in a plastic wrap immediately at birth without drying. This indication is based on studies comparing wrapping without drying vs. drying without wrapping (5,6), following previous investigations on plastic wrapping which were not carried out in the delivery room environment (7,8). However, the potential advantages of combining drying and wrapping were not explored. A previous study investigated the role of drying before wrapping in the thermal management of preterm infants, and found comparable temperatures after birth in infants wrapped after being dried and in those wrapped without drying (9). However, no information is currently available for very preterm infants, who are at higher risk of heat loss due to evaporation (10).

2.2 Purpose of the analyses

These analyses will compare two approaches of thermal management (plastic wrapping with drying vs. plastic wrapping without drying) for preventing heat loss at birth in very preterm infants. These analyses will be included in the clinical study report.

3 Study Objectives and Endpoints

3.1 Study Objectives

To compare two approaches of thermal management (plastic wrapping with drying vs. plastic wrapping without drying) for preventing heat loss at birth in very preterm infants.

3.2 Endpoints

Primary:

- To compare the proportion of neonates in the normal thermal range (temperature 36.5-37.5°C) at NICU admission.

Secondary:

- To compare the proportion of neonates with hypothermia (temperature <36.5°C) at NICU admission;
- To compare the proportion of neonates with moderate-severe hypothermia (temperature <36.0°C) at NICU admission;
- To compare the proportion of hyperthermic neonates (temperature >37.5°C) at NICU admission;
- To compare the temperature at 1 hour after NICU admission;
- To compare the proportion of intraventricular hemorrhage (all grades and grade III-IV);
- To compare the proportion of respiratory distress syndrome (RDS);
- To compare the proportion of late onset sepsis;
- To compare the proportion of bronchopulmonary dysplasia;
- To compare the mortality before hospital discharge.

4 Study Methods

4.1 General Study Design and Plan

The study is a multicenter, unblinded, randomized controlled trial. The study will be conducted at 21 Italian Level III hospitals (see list in the trial protocol). The participants will be very preterm infants (inclusion and exclusion criteria are listed in section 4.2). Participants will be randomly assigned to intervention (drying before plastic wrapping) or control (plastic wrapping without drying) arms (randomization is described in section 4.3). Participants will be followed-up until death or discharge.

4.2 Inclusion-Exclusion Criteria and General Study Population

Inborn infants fulfilling the following inclusion criteria will be eligible to participate in the study: inborn baby, estimated birth weight <1,500 g and/or gestational age ≤30+6 weeks, parental consent. Infants with major malformations (incompatible with sustained life or affecting the airways) will be excluded.

4.3 Randomization and Blinding

Participants will be randomly allocated to intervention or control arms in a 1:1 ratio according to a computer-generated, randomized sequence for each participating hospital. Infants of multiple pregnancies will be randomized as individuals. Randomization (with blocks of size 4-6) will be stratified by center. The randomized allocation will be concealed in double-enclosed, opaque, sealed, and sequentially numbered envelopes. In the delivery room/operating room, the next sequential randomization envelope will be opened only when the neonate will be considered eligible by the attending team, and the assigned procedure will be performed. Contamination between arms will not be allowed. Due to the characteristics of the intervention, neither caregivers nor outcome assessors will be masked to treatment allocation. However, the statistician who will perform data analysis will be blind for treatment allocation.

4.4 Study Variables

During the study, data will be collected about: patient anonymized identification and arm assignment; antenatal records (i.e. mother's age, pregnancy complications, ...); birth information (i.e. delivery mode, temperature of delivery/operation room …); contamination between arms (details and reasons); demographics (i.e. gestational age, birth weight, ..); thermal interventions; respiratory support; outcome measures associated with the endpoints; serious adverse events; discharge information.

5 Sample Size

Based on literature data (11), we hypothesize that the proportion of neonates in the normal thermal range at NICU admission may increase from 40% in the control arm to 55% in the intervention arm. With a power of 80% and an error I type of 0.05, the minimum sample size is estimated in 346 neonates (173 per arm). As the design is a stratified multicenter individually randomized trial, randomization is balanced and stratified on centers, and we expect no loss of power as a result of randomizing neonates by center (12). Sample size calculations were performed using R 4.1 software (R Foundation for Statistical Computing, Vienna, Austria) (13).

6 General Considerations

6.1 Timing of Analyses

The final analysis will be performed at the end of the enrolment or after stopping of the trial as suggested by the monitoring board.

6.2 Approach for main analysis

Data analysis will be performed using the statistical software packages R. All tests will be two-sided and a p-value less than 0.05 will be considered statistically significant. Statistical analysis will include an unadjusted analysis followed by an adjusted analysis.

Analysis will be performed according to the "intention to treat" (ITT) approach. In case of deviations from the protocol regarding the contamination between arms, a per-protocol analysis will be also performed, and the conclusion will be drawn according to the results of both analyses.

Additional sub-group analyses will be considered with exploratory purpose.

6.3 Additional analysis

A sub-analysis according to gestational age (23+0-27+6 and 28+0-31+6 weeks) will be performed with exploratory purpose.

6.4 Missing Data

Missing data will be considered, and appropriate imputations will be discussed (and performed when appropriate) with a multidisciplinary approach, in order to taken into account statistical, methodological and clinical aspects of this issue.
Missing data due to the premature discontinuation from the study or treatment are unlikely. Appropriate measures will be undertaken in order to avoid missing data, including regular double check of completeness of data forms and review of the video recordings.
Missing data will be described in the study report.

6.5 Interim Analyses and Data Monitoring

A monitoring board will review all deaths and adverse effects, as described in the trial protocol.
An interim analysis will be performed on the primary endpoint and the serious adverse events (SAEs) from the first 100 infants enrolled. The interim analysis will be performed by the statistician, blinded for the treatment allocation, who will report to the principal investigator. The principal investigator will discuss the results of the interim analysis with the monitoring board and the trial will be ended in case of harm. Criteria for stopping for harm include: a statistically significant difference ($p<0.001$ according to Haybittle-Peto boundary or $p<0.0294$ according to Pocock boundary) (14) in the primary outcome between the treatment groups; a reasonable suspected causal relationship between the intervention and SAEs.
Stopping for futility and/or interim analysis for sample size adjustment are not planned.

7 Summary of Study Data

Data will be summarized as mean with standard deviation or median and interquartile range (continuous data), or frequency with percentage (categorical data). Number of non-missing data, contaminations between arms and lost to follow-up will be reported. Summary of data will be presented in tables.

8 Analysis of primary outcome measure

The primary outcome measure is the proportion of neonates in the normal thermal range (temperature 36.5-37.5°C) at NICU admission. It will be summarized as frequency with percentage for each arm. In the unadjusted analysis, it will be compared between the two arms using Chi Square test or Fisher's test. The adjusted analysis will involve a generalized mixed-effect regression model including the trial arms (fixed effect), the participating center (random effect) and unbalanced participant characteristics at baseline (fixed effects). Relative risk with 95% confidence intervals for treatment vs. control arms will be calculated in both unadjusted and adjusted analyses, with a confidence interval higher than 1 indicating superiority of the intervention over the control.

9 Analysis of secondary outcome measures

The secondary outcome measures are presented in section 3.2.
Binary secondary outcome measures will be summarized as frequency with percentage for each arm. In the unadjusted analysis, they will be compared between the two treatment arms using Chi Square

test or Fisher's test. The adjusted analysis will involve a generalized mixed-effect regression model including the trial arms (fixed effect), the participating center (random effect) and unbalanced participant characteristics at baseline (fixed effects). Relative risk with 95% confidence intervals for treatment vs. control arms will be calculated in both unadjusted and adjusted analyses, with a confidence interval lower than 1 indicating superiority of the intervention over the control.
The only continuous secondary outcome measures will be summarized as mean (SD) or median (IQR) for each arm. In the unadjusted analysis, it will be compared between the two treatment arms using Student's t-test or Mann-Whitney test. The adjusted analysis will involve a linear mixed-effect regression model including the trial arms (fixed effect), the participating center (random effect) and unbalanced participant characteristics at baseline (fixed effects). Mean difference with 95% confidence intervals for treatment vs. control arms will be calculated in both unadjusted and adjusted analyses, with a confidence interval excluding 0 indicating a difference of the intervention vs. the control.

10. Exploratory Analyses

The outcome measures will be compared between the trial arms within strata of interest: participants with gestational age between 23^{+0} and 27^{+6} weeks, and participants with gestational age between 28 ^{and} 31^{+6} weeks). These analyses will be performed with exploratory purpose.

10 Safety Analyses

The trial protocol enlists unexpected death, severe hyperthermia (temperature >39°C) and hypothermia (temperature <35°C) as serious adverse events (SAEs). Occurrence of SAEs will be summarized as frequency with percentage and compared between arms using Chi Square test or Fisher's test.

11 Other Analyses

Additional analyses to be performed in the future will be discussed after the interim analysis and include in the SAP. Further analyses may subsequently be performed outside the scope of the SAP using the study data and published if appropriate, but they will be clearly described as post-hoc analyses.

13 Summary of Changes to the Protocol

The statistical analysis plan does not propose any changes to the statistical approach described in the referred protocol version (see front page).

14 References

1. Trevisanuto D, Sedin G. Thermal homeostasis and clinical management. In: Neonatology. A Practical Approach to Neonatal Diseases. Buonocore G, Bracci R, Weindling M. Springer Ed 2016.

2. Wilson E, Maier RF, Norman M, Misselwitz B, Howell EA, Zeitlin J, Bonamy AK; Effective Perinatal Intensive Care in Europe (EPICE) Research Group. Admission Hypothermia in Very Preterm Infants and Neonatal Mortality and Morbidity. J Pediatr. 2016;175:61-67.e4.
3. Wyckoff MH, Aziz K, Escobedo MB, Kapadia VS, Kattwinkel J, Perlman JM, Simon WM, Weiner GM, ZaichKin JG. Part 13: Neonatal Resuscitation: 2015 AHA Guidelines Update for CardiopulmonaryResuscitation and Emergency Cardiovascular Care. Circulation 2015; 132 (suppl 2):S543-S560.
4. Wyllie J, Bruinenberg J, Roehr CC, Rüdiger M, Trevisanuto D, Urlesberger B. European Resuscitation Council Guidelines for Resuscitation 2015: Section 7. Resuscitation and support of transition of babies at birth. Resuscitation. 2015;95:249-63.
5. Vohra S, Frent G, Campbell V, Abbott M, Whyte R. Effect of polyethylene occlusive skin wrapping on heat loss in very low birth weight infants at delivery: a randomized trial. J Pediatr. 1999 May;134(5):547-51.
6. McCall EM, Alderdice F, Halliday HL, Vohra S, Johnston L. Interventions to prevent hypothermia at birth in preterm and/or low birth weight infants. Cochrane Database Syst Rev. 2018;2(2):CD004210
7. Baumgart S, Engle WD, Fox WW, Polin RA. Effect of heat shielding on convective and evaporative heat losses and on radiant heat transfer in the premature infant. J Pediatr 1981;99:948-56
8. Baumgart S. Reduction of oxygen consumption, insensible water loss, and radiant heat demand with use of a plastic blanket for low-birth-weight infants under radiant warmers. Pediatrics 1984;74:1022-8.
9. Cardona Torres LM, Amador Licona N, Garcia Campos ML, Guizar-Mendoza JM. Polyethylene wrap for thermoregulation in the preterm infant: a randomized trial. Indian Pediatr. 2012 Feb;49(2):129-32.
10. Trevisanuto D, Testoni D, de Almeida MFB. Maintaining normothermia: Why and how? Semin Fetal Neonatal Med. 2018;23(5):333-339.
11. Cavallin F, Doglioni N, Allodi A, Battajon N, Vedovato S, Capasso L, Gitto E, Laforgia N, Paviotti G, Capretti MG, Gizzi C, Villani PE, Biban P, Pratesi S, Lista G, Ciralli F, Soffiati M, Staffler A, Baraldi E, Trevisanuto D; Servo COntrol for PReterm Infants (SCOPRI) Trial Group. Thermal management with and without servo-controlled system in preterm infants immediately after birth: a multicentre, randomised controlled study. Arch Dis Child Fetal Neonatal Ed. 2021 Nov;106(6):572-577.
12. Vierron E, Giraudeau B. Design effect in multicenter studies: gain or loss of power? BMC Med Res Methodol 2009;9:39.
13. R Core Team (2022). R: A language and environment for statistical computing. R Foundation for Statistical Computing, Vienna, Austria. URL https://www.R-project.org/.
14. Pocock SJ. When (not) to stop a clinical trial for benefit. JAMA 2005;294:2228–30.
