# Supplementary material for: Drying Very Preterm Infants Before Plastic Wrapping at Birth: A Randomized Clinical Trial
Source: JAMA Netw Open. 2026 Mar 3;9(3):e2556902. doi: 10.1001/jamanetworkopen.2025.56902 (PMC12958082; doi:10.1001/jamanetworkopen.2025.56902)
Supplement: Supplement 2. — eMethods. Description of the statistical methods eTable 1. Multivariable Analysis of Mortality eTable 2. Timing and Cause of Death of Deceased Neonates eTable 3. Information at NICU Admission of Deceased Neonates eTable 4. Outcome Measures in Neonates With Gestational Age Between 23+0 and 27+6 Weeks eTable 5. Outcome Measures in Neonates With Gestational Age Between 28+0 and 31+6 Weeks eTable 6. Adjusted Analysis of the Binary Outcome Measures by Using a Modified Poisson Model With Robust Standard Errors eTable 7. Adjusted Analysis of the Binary Outcome Measures by Using a Modified Poisson Model With Robust Standard Errors in Neonates With Gestational Age Between 23+0 and 27+6 Weeks eTable 8. Adjusted Analysis of the Binary Outcome Measures by Using a Modified Poisson Model With Robust Standard Errors in Neonates With Gestational Age Between 28+0 and 31+6 Weeks eTable 9. Multivariable Analysis of Mortality by Using a Modified Poisson Model With Robust Standard Errors eTable 10. Sensitivity Analysis With Adjustment for Multiple Testing Using Benjamini–Hochberg Procedure [file jamanetwopen-e2556902-s002.pdf]

Supplementary Online Content

Cavallin F, Doglioni N, Risso FM, et al; NEODRY Trial Group. Drying very preterm infants before plastic wrapping at birth: a randomized clinical trial. *JAMA Netw Open.* 2026;9(2):e2556902. doi:10.1001/jamanetworkopen.2025.56902

eMethods. Description of the statistical methods

eTable 1. Multivariable Analysis of Mortality

eTable 2. Timing and Cause of Death of Deceased Neonates

eTable 3. Information at NICU Admission of Deceased Neonates

eTable 4. Outcome Measures in Neonates With Gestational Age Between 23^{+0} and 27^{+6} Weeks

eTable 5. Outcome Measures in Neonates With Gestational Age Between 28^{+0} and 31^{+6} Weeks

eTable 6. Adjusted Analysis of the Binary Outcome Measures by Using a Modified Poisson Model With Robust Standard Errors

eTable 7. Adjusted Analysis of the Binary Outcome Measures by Using a Modified Poisson Model With Robust Standard Errors in Neonates With Gestational Age Between 23^{+0} and 27^{+6} Weeks

eTable 8. Adjusted Analysis of the Binary Outcome Measures by Using a Modified Poisson Model With Robust Standard Errors in Neonates With Gestational Age Between 28^{+0} and 31^{+6} Weeks

eTable 9. Multivariable Analysis of Mortality by Using a Modified Poisson Model With Robust Standard Errors

eTable 10. Sensitivity Analysis With Adjustment for Multiple Testing Using Benjamini–Hochberg Procedure

This supplementary material has been provided by the authors to give readers additional information about their work.

© 2026 Cavallin F et al. *JAMA Network Open*.

eMethods. Description of the Statistical Analysis

All analyses were performed according to the intention-to-treat approach. The trial protocol specified the option of adding a per-protocol analysis in case of contamination between arms, to draw the conclusions according to the results of both analyses. As all participants received the allocated intervention, only the intention-to-treat approach was applied.

Statistical significance was set at 5%. Statistical analysis was performed using R 4.4 software (R Foundation for Statistical Computing, Vienna, Austria).

An interim analysis was performed on the primary outcome measure and the serious adverse events in the first 100 enrolled participants. The criteria for stopping for harm included i) a statistically significant difference ($p<0.001$ according to Haybittle-Peto boundary) in the primary outcome between the treatment arms, or ii) a sensible supposed causal association between the intervention and the serious adverse events. Stopping criteria for futility and interim analyses for sample size adjustment were not planned. A group sequential approach with Haybittle-Peto boundary (which includes a $p<0.001$ threshold for the interim analysis and $p<0.05$ for the final analysis) was employed to prevent inflation of type I error.

Continuous data were summarized as mean and standard deviation, and categorical data as absolute and relative frequency (percentage). Missing data were few and limited to some patient characteristics hence a complete-case analysis was performed. The main analysis included both unadjusted and adjusted analyses.

In the unadjusted analysis, the outcome measures were compared between the two arms using Chi-square test or Fisher's test (categorical variables), or Student's t test (continuous variables).

The adjusted analysis employed Cox mixed-effects model with the time set at 1 (for binary outcome measures) and linear mixed-effect models (for continuous outcome measures) including the trial arms as fixed effect and the participating center as random effect. The trial protocol specified the option of including unbalanced participant characteristics at baseline as fixed effects, but there were none. Effect sizes were reported as relative risk (RR) or mean difference (MD) with 95% confidence interval (CI).

A pre-planned sub-analysis of the outcome measures stratified by gestational age (from $23^{+0}$to 27^{+6} weeks and from 28^{+0} to 31^{+6} weeks) was performed with exploratory purpose.

A post-hoc multivariable analysis of mortality was carried out by including the trial arms and clinically relevant confounders as independent variables in the regression model.

A post-hoc sensitivity analysis replicated the adjusted analysis of the binary outcome measures by using a modified Poisson model with robust standard errors.

A post-hoc sensitivity analysis adjusted for multiple testing all comparisons of the outcome measures between dried and undried arms by using Benjamini–Hochberg procedure.

© 2026 Cavallin F et al. *JAMA Network Open*.

eTable 1. Multivariable Analysis of Mortality

Independent variable	Model #1 (including the intervention and clinically relevant factors)		Model #2 (including the intervention and pre-intervention clinically relevant factors)	
	Risk ratio (95% confidence interval)	p-value	Risk ratio (95% confidence interval)	p-value
Drying vs. not drying before plastic wrapping	3.08 (1.44 to 6.56)	0.003	3.14 (1.50 to 6.58)	0.002
Sex: male vs. female	1.33 (0.65 to 2.68)	0.43	1.44 (0.72 to 2.89)	0.29
Gestational age, weeks	0.56 (0.46 to 0.69)	<0.0001	0.57 (0.49 to 0.66)	<0.0001
Multiple birth: yes vs. no	0.85 (0.36 to 2.05)	0.73	0.82 (0.34 to 1.95)	0.65
Intrauterine growth restriction (IUGR): yes vs. no	1.14 (0.31 to 4.12)	0.84	1.23 (0.35 to 4.38)	0.75
Intubation in delivery room: yes vs. no	0.72 (0.30 to 1.75)	0.47	-	-
Temperature at NICU admission, °C	0.76 (0.48 to 1.19)	0.23	-	-

© 2026 Cavallin F et al. *JAMA Network Open*.

eTable 2. Timing and Cause of Death of Deceased Neonates

Number	Arm	Timing of the death	Comment
1	Drying	Late mortality (>48 hours)	BPD in extremely preterm infant (25-27 weeks' gestation)
2	Drying	Early mortality (≤48 hours)	IVH in extremely preterm infant (23 weeks' gestation)
3	Drying	Late mortality (>48 hours)	Severe IVH in extremely preterm infant (23 weeks' gestation)
4	Drying	Early mortality (≤48 hours)	Severe IVH in extremely preterm infant (23 weeks' gestation)
5	Drying	Late mortality (>48 hours)	Extremely preterm infant (25-27 weeks' gestation)
6	Drying	Late mortality (>48 hours)	NEC in extremely preterm infant (25-27 weeks' gestation)
7	Drying	Late mortality (>48 hours)	Extremely preterm infant (25-27 weeks' gestation)
8	Drying	Early mortality (≤48 hours)	Extremely preterm infant (23 weeks' gestation)
9	Drying	Late mortality (>48 hours)	IVH in extremely preterm infant (25-27 weeks' gestation)
10	Drying	Late mortality (>48 hours)	Severe IVH in extremely preterm infant (25-27 weeks' gestation)
11	Drying	Early mortality (≤48 hours)	Extremely preterm infant (25-27 weeks' gestation)
12	Drying	Late mortality (>48 hours)	Severe IVH in extremely preterm infant (23 weeks' gestation)
13	Drying	Late mortality (>48 hours)	Severe IVH, LOS and NEC in extremely preterm infant (25-27 weeks' gestation)
14	Drying	Early mortality (≤48 hours)	Severe IVH in very preterm infant (28-30 weeks' gestation)
15	Drying	Late mortality (>48 hours)	LOS and BPD in extremely preterm infant (24 weeks' gestation)
16	Drying	Late mortality (>48 hours)	IVH in extremely preterm infant (24 weeks' gestation)
17	Drying	Early mortality (≤48 hours)	IUGR in very preterm infant (28-30 weeks' gestation)
18	Drying	Late mortality (>48 hours)	Extremely preterm infant (25-27 weeks' gestation)
19	Drying	Late mortality (>48 hours)	Severe IVH in very preterm infant (28-30 weeks' gestation)
20	Drying	Early mortality (≤48 hours)	Severe IVH in extremely preterm infant (23 weeks' gestation)
21	Drying	Late mortality (>48 hours)	BPD in extremely preterm infant (25-27 weeks' gestation)
22	Drying	Late mortality (>48 hours)	LOS in extremely preterm infant (25-27 weeks' gestation)
23	Drying	Late mortality (>48 hours)	BPD and LOS in extremely preterm infant (25-27 weeks' gestation)
24	Drying	Late mortality (>48 hours)	Severe IVH and LOS in extremely preterm infant (25-27 weeks' gestation)
25	Drying	Early mortality (≤48 hours)	Extremely preterm infant (23 weeks' gestation)
26	Drying	Late mortality (>48 hours)	LOS in very preterm infant (28-30 weeks' gestation)
27	Not drying	Late mortality (>48 hours)	IVH and LOS in very preterm infant (28-30 weeks' gestation)
28	Not drying	Late mortality (>48 hours)	LOS in extremely preterm infant (24 weeks' gestation)
29	Not drying	Late mortality (>48 hours)	IVH in extremely preterm infant (24 weeks' gestation)
30	Not drying	Late mortality (>48 hours)	Severe IVH in extremely preterm infant (24 weeks' gestation)
31	Not drying	Early mortality (≤48 hours)	Severe IVH in very preterm infant (28-30 weeks' gestation)
32	Not drying	Early mortality (≤48 hours)	Very preterm infant (28-30 weeks' gestation)
33	Not drying	Early mortality (≤48 hours)	Severe IVH in extremely preterm infant (23 weeks' gestation)
34	Not drying	Early mortality (≤48 hours)	Severe IVH in extremely preterm infant (23 weeks' gestation)
35	Not drying	Late mortality (>48 hours)	Extremely preterm infant (25-27 weeks' gestation)
36	Not drying	Late mortality (>48 hours)	Severe IVH in extremely preterm infant (24 weeks' gestation)

BPD, bronchopulmonary dysplasia; IVH, intraventricular hemorrhage; LOS, late onset sepsis; NEC, neonatal enterocolitis. Severe IVH included grades III-IV.

© 2026 Cavallin F et al. *JAMA Network Open*.

eTable 3. Information at NICU Admission of Deceased Neonates

Number	Arm	Age, minutes	Hypothermia	Max respiratory support	pH	pCO2, mmHg	pO2, mmHg	HCO3, mmol/l	BE, mmol/l	Lactates, mmol/l	Glycemia, mg/dl
1	A	12	no	CPAP	7.41	37.6	-	23.6	-0.9	5.3	39
2	A	30	no	Intubation	7.21	48.2	67.4	19.2	-8.6	6	48
3	A	15	no	Intubation	7.29	37.5	85.3	17.6	-9	5.7	48
4	A	20	yes	Intubation	6.9	84	-	16	17	6.5	90
5	A	21	no	CPAP	7.32	47.8	57.7	24.1	-2.5	3	21
6	A	26	yes	Intubation	7.21	41.3	103.5	16.5	-11.7	11.98	NA
7	A	11	yes	Intubation	7.34	30	56	18.1	-8.4	8.1	53
8	A	1	yes	Intubation	7.33	37.3	15.9	20.2	-5.8	5.1	126
9	A	27	yes	Intubation	7.1	53.5	63	16	-13	11.02	28
10	A	25	yes	Intubation	6.99	89	51	15	-10.3	0.9	226
11	A	23	yes	Intubation	7.21	51.6	32.4	17.6	-7.6	2.02	54
12	A	34	yes	Intubation	7.04	31.7	116.3	9.5	-21.9	5.85	115
13	A	55	yes	Intubation	7.02	82.5	55.8	16	-9.1	4.2	57
14	A	33	yes	CPAP	7.04	93	53	25.1	-7.7	1.1	30
15	A	27	no	CPAP	7.251	54.9	97.6	21.2	-3.9	4.3	60
16	A	29	no	Intubation	7.198	51.9	42.3	17.7	-8.1	3.7	89
17	A	21	yes	Intubation	7.23	53.7	52.7	19.7	-5.6	4.9	45
18	A	40	yes	Intubation	7.486	27.9	37.3	23	-2.3	2.4	40
19	A	34	yes	CPAP	7.27	43	40	19.7	-7.2	1.5	97
20	A	22	no	Intubation	7.34	26	47	14	-12	6.3	50
21	A	23	no	Intubation	7.03	76	49	20.1	-11.1	7	81
22	A	23	yes	CPAP	7.31	50	70	25.2	-1.6	3.4	86
23	A	20	yes	CPAP	7.32	45.5	32.9	23.2	-2.9	2.01	58
24	A	33	yes	Intubation	7.39	31	50	20.7	-5	2.6	25
25	A	44	yes	Intubation	7.25	43.4	30.4	17.2	-8.4	3	38
26	A	20	yes	Intubation	7.16	68.8	53.7	19.4	-4	0.9	50
27	B	26	no	CPAP	7.29	52.2	41.9	24.9	-2.2	2.2	25
28	B	10	yes	CPAP	7.39	32	72	19.4	-5.6	5.5	60
29	B	29	no	Intubation	7.28	32	90	15	-11	5.4	33
30	B	1	no	Intubation	7.37	39	22	22.5	-2.8	5.3	46
31	B	25	yes	CPAP	7.29	50	93	22.4	-2.6	1.1	26
32	B	27	yes	CPAP	7.4	37	35	23.1	-1.3	2	89
33	B	21	yes	Intubation	7.34	40	23	20.7	-4.2	2.9	69
34	B	23	yes	Intubation	7.42	36	41	24	-1.1	2.1	80
35	B	3	yes	CPAP	7.206	63.2	6.2	18.6	-4.4	3	76
36	B	14	no	CPAP	7.24	54.9	45.7	23.1	-4.8	1.98	38

© 2026 Cavallin F et al. *JAMA Network Open*.

eTable 4. Outcome Measures in Neonates With Gestational Age Between 23^{+0} and 27^{+6} Weeks

Primary and secondary outcomes	Outcome measure	Drying before plastic wrapping (n=47)	Not drying before plastic wrapping (n=55)	Analysis adjusted for centre	
		N (%) or mean (SD)	N (%) or mean (SD)	RR (95% CI) or MD (95% CI)	p-value
Primary outcome measure	Normothermia (36.5-37.5°C)	19 (40%)	25 (45%)	0.85 (0.46 to 1.56)	0.60
Secondary outcome measures	Hypothermia (<36.5°C	27 (57%)	27 (49%)	1.40 (0.81 to 2.44)	0.23
	Moderate-severe hypothermia (<36.0°C)	12 (28%)	12 (22%)	1.43 (0.64 to 3.22)	0.39
	Hyperthermia (>37.5°C)	1 (2%)	(5%)	0.35 (0.04 to 3.44)	0.37
	Temperature after 1 hour, °C	36.1 (1.0)	36.4 (0.7)	-0.3 (-0.6 to -0.1)	0.04
	IVH (all grades)	19/46 (41%)	20 (36%)	1.17 (0.63 to 2.20)	0.62
	IVH (III-IV grades)	9/46 (20%)	8 (15%)	1.38 (0.53 to 3.57)	0.51
	RDS	36/46 (78%)	41 (75%)	1.11 (0.70 to 1.75)	0.66
	Late onset sepsis	14/46 (30%)	23 (42%)	0.70 (0.36 to 1.39)	0.32
	BPD	19/40 (47%)	27/54 (50%)	1.08 (0.59 to 2.00)	0.77
	Mortality	22 (47%)	7 (13%)	4.71 (2.00 to 11.12)	0.0004

BPD, bronchopulmonary dysplasia; CI: confidence interval; IVH, intraventricular hemorrhage; MD: mean difference; RDS, respiratory distress syndrome; RR, relative risk.

© 2026 Cavallin F et al. *JAMA Network Open*.

eTable 5. Outcome Measures in Neonates With Gestational Age Between 28^{+0} and 31^{+6} Weeks

Primary and secondary outcomes	Outcome measure	Drying before plastic wrapping (n=107)	Not drying before plastic wrapping (n=104)	Analysis adjusted for centre	
		N (%) or mean (SD)	N (%) or mean (SD)	RR (95% CI) or MD (95% CI)	p-value
Primary outcome measure	Normothermia (36.5-37.5°C)	52 (48%)	50 (48%)	1.05 (0.71 to 1.55)	0.82
Secondary outcome measures	Hypothermia (<36.5°C	51 (47%)	44 (42%)	1.21 (0.81 to 1.82)	0.35
	Moderate-severe hypothermia (<36.0°C)	27 (24%)	18 (17%)	1.52 (0.83 to 2.79)	0.18
	Hyperthermia (>37.5°C)	6 (5%)	10 (10%)	0.56 (0.20 to 1.55)	0.27
	Temperature after 1 hour, °C	36.3 (0.7)	36.4 (0.5)	-0.1 (-0.2 to 0.1)	0.40
	IVH (all grades)	11/106 (10%)	14 (13%)	0.73 (0.33 to 1.62)	0.44
	IVH (III-IV grades)	5/106 (5%)	6 (6%)	0.80 (0.24 to 2.62)	0.71
	RDS	86 (79%)	84 (81%)	0.96 (0.71 to 1.29)	0.77
	Late onset sepsis	15 (14%)	14 (13%)	1.02 (0.49 to 2.12)	0.95
	BPD	23/104 (22%)	23/102 (23%)	0.86 (0.48 to 1.54)	0.62
	Mortality	4 (4%)	3 (3%)	1.19 (0.26 to 5.31)	0.82

BPD, bronchopulmonary dysplasia; CI: confidence interval; IVH, intraventricular hemorrhage; MD: mean difference; RDS, respiratory distress syndrome; RR, relative risk.

© 2026 Cavallin F et al. *JAMA Network Open*.

eTable 6. Adjusted Analysis of the Binary Outcome Measures by Using a Modified Poisson Model With Robust Standard Errors

Binary outcome measure	Drying before plastic wrapping (n=177)	Not drying before plastic wrapping (n=177)	Unadjusted analysis		Analysis adjusted for center	
	N (%)	N (%)	RR (95% CI)	p-value	RR (95% CI)	p-value
Normothermia (36.5-37.5°C)	81 (45.8%)	82 (46.3%)	0.99 (0.79 to 1.24)	0.99	0.99 (0.78 to 1.24)	0.92
Hypothermia (<36.5°C	89 (50.3%)	81 (45.8%)	1.10 (0.88 to 0.36)	0.46	1.10 (0.92 to 1.31)	0.29
Moderate-severe hypothermia (<36.0°C)	40 (22.6%)	34 (19.2%)	1.18 (0.78 to 1.77)	0.51	1.18 (0.83 to 1.67)	0.36
Hyperthermia (>37.5°C)	7 (4.0%)	14 (7.9%)	0.50 (0.21 to 1.21)	0.18	0.50 (0.26 to 0.95)	0.03
IVH (all grades)	30/175 (17.1%)	35 (19.8%)	0.87 (0.56 to 1.35)	0.62	0.87 (0.63 to 1.18)	0.37
IVH (III-IV grades)	14/175 (8.0%)	14 (7.9%)	1.01 (0.50 to 2.06)	0.99	1.01 (0.44 to 2.33)	0.98
RDS	137/176 (77.8%)	139 (78.5%)	0.99 (0.89 to 1.11)	0.98	0.99 (0.89 to 1.10)	0.87
Late onset sepsis	32/176 (18.2%)	38 (21.5%)	0.85 (0.56 to 1.29)	0.52	0.85 (0.55 to 1.29)	0.44
BPD	44/167 (26.3%)	52/174 (29.9%)	0.88 (0.63 to 1.24)	0.54	0.88 (0.63 to 1.24)	0.45
Mortality	26 (14.7%)	10 (5.6%)	2.60 (1.29 to 5.23)	0.008	2.60 (0.99 to 6.76)	0.05

BPD, bronchopulmonary dysplasia; CI: confidence interval; IVH, intraventricular hemorrhage; RDS, respiratory distress syndrome; RR, relative risk.

© 2026 Cavallin F et al. *JAMA Network Open*.

eTable 7. Adjusted Analysis of the Binary Outcome Measures by Using a Modified Poisson Model With Robust Standard Errors in Neonates With Gestational Age Between 23^{+0} and 27^{+6} Weeks

Binary outcome measure	Drying before plastic wrapping (n=47)	Not drying before plastic wrapping (n=55)	Analysis adjusted for centre	
	N (%)	N (%)	RR (95% CI)	p-value
Normothermia (36.5-37.5°C)	19 (40%)	25 (45%)	0.89 (0.52 to 1.51)	0.66
Hypothermia (<36.5°C	27 (57%)	27 (49%)	1.17 (0.79 to 1.73)	0.43
Moderate-severe hypothermia (<36.0°C)	12 (28%)	12 (22%)	1.27 (0.69 to 2.14)	0.44
Hyperthermia (>37.5°C)	1 (2%)	(5%)	0.39 (0.08 to 1.87)	0.24
IVH (all grades)	19/46 (41%)	20 (36%)	1.14 (0.70 to 1.84)	0.60
IVH (III-IV grades)	9/46 (20%)	8 (15%)	1.34 (0.44 to 4.08)	0.60
RDS	36/46 (78%)	41 (75%)	1.05 (0.86 to 1.28)	0.64
Late onset sepsis	14/46 (30%)	23 (42%)	0.73 (0.42 to 1.26)	0.26
BPD	19/40 (47%)	27/54 (50%)	0.95 (0.63 to 1.43)	0.81
Mortality	22 (47%)	7 (13%)	3.68 (1.35 to 9.98)	0.01

BPD, bronchopulmonary dysplasia; CI: confidence interval; IVH, intraventricular hemorrhage; RDS, respiratory distress syndrome; RR, relative risk.

© 2026 Cavallin F et al. *JAMA Network Open*.

eTable 8. Adjusted Analysis of the Binary Outcome Measures by Using a Modified Poisson Model With Robust Standard Errors in Neonates With Gestational Age Between 28^{+0} and 31^{+6} Weeks

Binary outcome measure	Drying before plastic wrapping (n=107)	Not drying before plastic wrapping (n=104)	Analysis adjusted for centre	
	N (%)	N (%)	RR (95% CI)	p-value
Normothermia (36.5-37.5°C)	52 (48%)	50 (48%)	0.99 (0.76 to 1.30)	0.96
Hypothermia (<36.5°C	51 (47%)	44 (42%)	1.10 (0.89 to 1.38)	0.37
Moderate-severe hypothermia (<36.0°C)	27 (24%)	18 (17%)	1.38 (0.84 to 2.25)	0.20
Hyperthermia (>37.5°C)	6 (5%)	10 (10%)	0.57 (0.22 to 1.48)	0.25
IVH (all grades)	11/106 (10%)	14 (13%)	0.76 (0.46 to 1.24)	0.27
IVH (III-IV grades)	5/106 (5%)	6 (6%)	0.80 (0.23 to 2.75)	0.73
RDS	86 (79%)	84 (81%)	0.98 (0.87 to 1.10)	0.70
Late onset sepsis	15 (14%)	14 (13%)	1.02 (0.52 to 2.00)	0.95
BPD	23/104 (22%)	23/102 (23%)	0.96 (0.65 to 1.43)	0.85
Mortality	4 (4%)	3 (3%)	1.27 (0.33 to 4.86)	0.72

BPD, bronchopulmonary dysplasia; CI: confidence interval; IVH, intraventricular hemorrhage; RDS, respiratory distress syndrome; RR, relative risk.

© 2026 Cavallin F et al. *JAMA Network Open*.

eTable 9. Multivariable Analysis of Mortality by Using a Modified Poisson Model With Robust Standard Errors

Independent variable	Model #1 (including the intervention and clinically relevant factors)		Model #2 (including the intervention and pre-intervention clinically relevant factors)	
	Risk ratio (95% confidence interval)	p-value	Risk ratio (95% confidence interval)	p-value
Drying vs. not drying before plastic wrapping	2.39 (1.01 to 5.64)	0.04	2.45 (1.09 to 5.15)	0.03
Sex: male vs. female	1.30 (0.81 to 2.08)	0.28	1.36 (0.86 to 2.15)	0.18
Gestational age, weeks	0.61 (0.51 to 0.73)	<0.0001	0.61 (0.53 to 0.71)	<0.0001
Multiple birth: yes vs. no	0.82 (0.42 to 1.61)	0.57	0.79 (0.41 to 1.54)	0.50
Intrauterine growth restriction (IUGR): yes vs. no	1.15 (0.41 to 3.24)	0.79	1.21 (0.46 to 3.18)	0.70
Intubation in delivery room: yes vs. no	0.87 (0.51 to 1.46)	0.59	-	-
Temperature at NICU admission, °C	0.88 (0.72 to 1.07)	0.20	-	-

© 2026 Cavallin F et al. *JAMA Network Open*.

eTable 10. Sensitivity Analysis With Adjustment for Multiple Testing Using Benjamini–Hochberg Procedure

Analysis adjusted for center	Outcome measure	Comparison of drying vs. not drying before plastic wrapping: p-value adjusted according to Benjamini–Hochberg
Main analysis	Normothermia (36.5-37.5°C)	0.98
	Hypothermia (<36.5°C	0.79
	Moderate-severe hypothermia (<36.0°C)	0.79
	Hyperthermia (>37.5°C)	0.61
	Temperature after 1 hour, °C	0.67
	IVH (all grades)	0.89
	IVH (III-IV grades)	0.98
	RDS	0.95
	Late onset sepsis	0.79
	BPD	0.79
	Mortality	0.12
Multivariable analysis of mortality	Mortality (including the intervention and clinically relevant factors)	0.07
	Mortality (including the intervention and pre-intervention clinically relevant factors)	0.07
Sub-analysis in neonates with gestational age between 23^{+0} and 27^{+6} weeks	Normothermia (36.5-37.5°C)	0.92
	Hypothermia (<36.5°C	0.79
	Moderate-severe hypothermia (<36.0°C)	0.79
	Hyperthermia (>37.5°C)	0.79
	Temperature after 1 hour, °C	0.30
	IVH (all grades)	0.92
	IVH (III-IV grades)	0.88
	RDS	0.92
	Late onset sepsis	0.79
	BPD	0.95
	Mortality	0.03
Sub-analysis in neonates with gestational age between 28^{+0} and 31^{+6} weeks	Normothermia (36.5-37.5°C)	0.95
	Hypothermia (<36.5°C	0.79
	Moderate-severe hypothermia (<36.0°C)	0.79
	Hyperthermia (>37.5°C)	0.79
	Temperature after 1 hour, °C	0.79
	IVH (all grades)	0.79
	IVH (III-IV grades)	0.94
	RDS	0.95
	Late onset sepsis	0.98
	BPD	0.92
	Mortality	0.95
Sensitivity analysis of the binary outcome measures by using a modified Poisson model with robust standard errors	Normothermia (36.5-37.5°C)	0.98
	Hypothermia (<36.5°C	0.79
	Moderate-severe hypothermia (<36.0°C)	0.79
	Hyperthermia (>37.5°C)	0.29
	IVH (all grades)	0.79
	IVH (III-IV grades)	0.98
	RDS	0.97
	Late onset sepsis	0.79
	BPD	0.79
	Mortality	0.34
Sensitivity analysis of the binary outcome measures by using a modified Poisson model with robust standard errors in neonates with gestational age between 23^{+0} and 27^{+6} weeks	Normothermia (36.5-37.5°C)	0.92
	Hypothermia (<36.5°C	0.79
	Moderate-severe hypothermia (<36.0°C)	0.98
	Hyperthermia (>37.5°C)	0.79
	IVH (all grades)	0.79
	IVH (III-IV grades)	0.61
	RDS	0.67
	Late onset sepsis	0.89
	BPD	0.98
	Mortality	0.95
Sensitivity analysis of the binary outcome measures by	Normothermia (36.5-37.5°C)	0.79
	Hypothermia (<36.5°C	0.79

© 2026 Cavallin F et al. *JAMA Network Open*.

using a modified Poisson model with robust standard errors in neonates with gestational age between 28^{+0} and 31^{+6} weeks	Moderate-severe hypothermia (<36.0°C)	0.12
	Hyperthermia (>37.5°C)	0.07
	IVH (all grades)	0.07
	IVH (III-IV grades)	0.92
	RDS	0.79
	Late onset sepsis	0.79
	BPD	0.79
	Mortality	0.30
Multivariable analysis of mortality by using a modified Poisson model with robust standard errors	Mortality (including the intervention and clinically relevant factors)	0.92
	Mortality (including the intervention and pre-intervention clinically relevant factors)	0.88

© 2026 Cavallin F et al. *JAMA Network Open*.
