# Supplementary material for: Drying Very Preterm Infants Before Plastic Wrapping at Birth: A Randomized Clinical Trial
Source: JAMA Netw Open. 2026 Mar 3;9(3):e2556902. doi: 10.1001/jamanetworkopen.2025.56902 (PMC12958082; doi:10.1001/jamanetworkopen.2025.56902)
Supplement: Supplement 3. — Nonauthor Collaborators [file jamanetwopen-e2556902-s003.pdf]

Supplemental Online Content: Nonauthor Collaborators
*First name, last name, and suffix (if applicable) are required and will appear in PubMed.

*Group Name(s): NEODRY Trial Group

*First Name and Middle Initial(s)	*Last Name	*Suffix (eg, Jr, III)	Academic Degrees	Institution	Location (city, state/province, country)	Role or Contribution, eg, chair, principal investigator	Group (if more than 1 Group listed in the byline) and/or Subgroup (eg, Steering Committee)
Eleonora	Gerardini		MD	Neonatal Intensive Care Unit, Dipartimento Materno Infantile, Sa	Perugia, Italy	Data collection and critically review of the manuscript	The NEODRY-TRIAL Group
Valeria	Manfredini		MD	Department of Pediatrics, Ospedale dei Bambini “V.Buzzi”	Milano, Italy	Data collection and critically review of the manuscript	The NEODRY-TRIAL Group
Paola	Lago			Critical Care Department, Ospedale Regionale Ca Foncello	Treviso, Italy	Data collection and critically review of the manuscript	The NEODRY-TRIAL Group
Giovanni	Russo		MD	UOC Neonatologia e TIN Dipartimento Materno-Infantile AORN M	Avellino, Italy	Data collection and critically review of the manuscript	The NEODRY-TRIAL Group
Genny	Gottardi		MD	Neonatal Intensive Care Unit, San Bortolo Hospital	Vicenza, Italy	Data collection and critically review of the manuscript	The NEODRY-TRIAL Group
Antonia	Filannino		MD	Neonatology and Neonatal Intensive Care Unit, Department of In	Bari, Italy	Data collection and critically review of the manuscript	The NEODRY-TRIAL Group
Giovanni	Vento		MD	Neonatal Intensive Care Unit, Department of Woman and Child H	Roma, Italy	Data collection and critically review of the manuscript	The NEODRY-TRIAL Group
Maria Francesca	Campagnoli		MD	Neonatal Intensive Care Unit, Sant’Anna Hospital, Città della Salu	Torino, Italy	Data collection and critically review of the manuscript	The NEODRY-TRIAL Group
Lucia	Marseglia		MD	Department of Pediatrics, University of Messina, Neonatal Intens	Messina, Italy	Data collection and critically review of the manuscript	The NEODRY-TRIAL Group
Alberto	Berardi		MD	Neonatal Intensive Care Unit, Policlinico University Hospital	Modena, Italy	Data collection and critically review of the manuscript	The NEODRY-TRIAL Group
Renzo	Beghini		MD	Department of Pediatrics, NICU, University Hospital of Verona	Verona, Italy	Data collection and critically review of the manuscript	The NEODRY-TRIAL Group
Mary	Velletri		MD	Neonatologia e TIN, Grande Ospedale Metropolitano	Reggio Calabria, Italy	Data collection and critically review of the manuscript	The NEODRY-TRIAL Group
