# Supplementary material for: Drying Very Preterm Infants Before Plastic Wrapping at Birth: A Randomized Clinical Trial
Source: JAMA Netw Open. 2026 Mar 3;9(3):e2556902. doi: 10.1001/jamanetworkopen.2025.56902 (PMC12958082; doi:10.1001/jamanetworkopen.2025.56902)
Supplement: Supplement 4. — Data Sharing Statement [file jamanetwopen-e2556902-s004.pdf]

Data Sharing Statement

Cavallin. Drying Very Preterm Infants Before Plastic Wrapping at Birth. *JAMA Netw Open*. Published February 16, 2026. doi:10.1001/jamanetworkopen.2025.56902

Data
Additional Information: ClinicalTrial.gov NCT05740072
Data available: Yes
Data types: Deidentified participant data
How to access data: Provide request for data must be sent to an individual: Daniele Trevisanuto daniele.trevisanuto@unipd.it
When available: With publication

Supporting Documents
Document types: Statistical/analytic code
How to access documents: daniele.trevisanuto@unipd.it
When available: With publication

Additional Information
Who can access the data: daniele.trevisanuto@unipd.it
Types of analyses: For a specific purpose
Mechanisms of data availability: With investigator support and with a signed data access agreement
